# miR34a-5p impedes CLOCK expression in chronodisruptive C57BL/6J mice and potentiates pro-atherogenic manifestations

**DOI:** 10.1371/journal.pone.0283591

**Published:** 2023-08-10

**Authors:** Hitarthi Vyas, Aliasgar Vohra, Kapil Upadhyay, Menaka Thounaojam, Ravirajsinh Jadeja, Nilay Dalvi, Manuela Bartoli, Ranjitsinh Devkar

**Affiliations:** 1 Division of Metabolic Endocrinology, Department of Zoology, Faculty of Science, The Maharaja Sayajirao University of Baroda, Vadodara, India; 2 Department of Internal Medicine, University of Michigan, Ann Arbor, MI, United States of America; 3 Department of Biochemistry and Molecular Biology, Augusta University, Augusta, GA, United States of America; 4 Department of Ophthalmology, Medical College of Georgia, Augusta University, Augusta, GA, United States of America; Universita Politecnica delle Marche, ITALY

## Abstract

**Introduction:**

Altered circadian rhythms underlie manifestation of several cardiovascular disorders, however a little is known about the mediating biomolecules. Multiple transcriptional-translational feedback loops control circadian-clockwork wherein; micro RNAs (miRNAs) are known to manifest post transcriptional regulation. This study assesses miR34a-5p as a mediating biomolecule.

**Method:**

8–10-week-old male C57BL/6J mice (n = 6/group) were subjected to photoperiodic manipulation induced chronodisruption and thoracic aortae were examined for miRNA, gene (qPCR) and protein (Immunoblot) expression studies. Histomorphological changes were assessed for pro-atherogenic manifestations (fibrillar arrangement, collagen/elastin ratio, intima-media thickening). Computational studies for miRNA-mRNA target prediction were done using TargetScan and miRDB. Correlative in vitro studies were done in serum synchronized HUVEC cells. Time point based studies were done at five time points (ZT 0, 6, 12, 18, 24) in 24h.

**Results:**

Chronodisruption induced hypomethylation in the promoter region of miR34a-5p, in the thoracic aortae, culminating in elevated miRNA titers. In a software-based detection of circadian-clock-associated targets of miR34a-5p, *Clock* and *Sirt1* genes were identified. Moreover, miR34a-5p exhibited antagonist circadian oscillations to that of its target genes CLOCK and SIRT1 in endothelial cells. Luciferase reporter gene assay further showed that miR34a-5p interacts with the 3’UTR of the *Clock* gene to lower its expression, disturbing the operation of positive arm of circadian clock system. Elevated miR34a-5p and impeded SIRT1 expression in a chronodisruptive aortae exhibited pro-atherogenic changes observed in form of gene expression, increased collagen/elastin ratio, fibrillar derangement and intimal-media thickening.

**Conclusion:**

The study reports for the first time chronodisruption mediated miR34a-5p elevation, its circadian expression and interaction with the 3’UTR of *Clock* gene to impede its expression. Moreover, elevated miR34a-5p and lowered SIRT1 expression in the chronodisruptive aortae lead off cause-consequence relationship of chronodisruption mediated proatherogenic changes.

## Introduction

Circadian clock setup has internally driven 24-hour rhythm, entraining to the external environmental day-night cycles. Circadian rhythms entrain several pathophysiological processes and viceversa with a complex molecular framework, functioning in synergy. Core clock genes CLOCK (Circadian Locomotor Output Cycles Kaput) and BMAL1 (aryl hydrocarbon receptor nuclear translocator like 1) acts as the key regulators of positive arm of circadian clockwork that initiates transcription of clock-controlled genes (CCGs; *Per1*, *Per2*, *Cry1*, *Cry2*) [1, 2]. In the negative arm, PER-CRY complex translocate into the nucleus and inhibit the CLOCK/BMAL-1 mediated gene transcription, completing the negative feedback loop [3]. Circadian rhythms are operated via multiple interactive transcriptional-translational feedback loops that integrate circadian rhythm of the body to its physiology. One such critical gene is SIRT1 (Sirtuin1) that functions at a chrono-metabolic juncture. Along with maintaining metabolic homeostasis in the tissue, SIRT1 deacetylates BMAL1 to facilitate CLOCK–BMAL1 heterodimerization that is responsible for activation of the positive arm of circadian clock system [4]. Along with gene regulations a major contributor regulating chrono-metabolic processes is micro-RNAs (miRNAs) that have been instituted as biological fine-tuners. Several studies have reported miRNA mediated fine tuning of circadian oscillation, phase shifts and circadian gene expressions [5–7].

miRNAs are small non-coding RNAs (20–22 single stranded nucleotides sequence) that regulates gene expression by translational repression and/or mRNA degradation by binding to 3’ untranslated region (UTR) of specific genes exhibiting complementary seed sequence. Mechanistically, miRNA can bind to multiple mRNAs, exhibiting complementary seed sequence, subjected to mRNA’s expressional availability, cellular location, and free energy of binding, implying towards pronounced regulatory impact. About 60% of the protein coding genes are regulated by miRNAs, demonstrating vital implications of miRNA in various physiological processes [8, 9]. miRNAs have been reported to elicit regulatory control on clock genes viz. *Clock* (miR17-5p), *Bmal1* (miR-191), *Per1* (miR34a), *Per2* (miR-25, miR34a) etc. [7, 10–13]. Study on the Circadian ‘proteome’ had revealed that the portion of cycling protein is rather higher than that of their ‘transcriptome’, and that many of the cycling proteins show constant abundance at the mature transcript level [14].

Alterations in circadian rhythms are known to influence pro-atherogenic changes in vascular endothelium and progression of the disease. About 330 genes (5% to 10% of the transcriptome) related to the core molecular clock, lipid and glucose metabolism, protein folding, and vascular integrity exhibit a circadian pattern in mouse aortae [15]. Loss of circadian rhythms (chronodisruption) has been reported as a major contributing factor for onset and progression of atherosclerosis [15], a dominant cause of several cardiovascular disorders. miRNA mediated regulation is well documented in initiation and progression of atherogenic pathology. Amongst modulated miRNAs, the expression of endothelial miR-21, miR-34a, and miR-146a, was found to be significantly upregulated in atherosclerotic arteries downregulating numerous predicted targets genes [16]. Several miRNAs viz miR217, miR34a, miR155 have been shown to inhibit expression of SIRT1 to manifest pro-atherogenic impact orchestrated in varied cell types [17–19]. Loss of SIRT1 expression in endothelial cells promotes inflammatory changes, expression of adhesion molecules, mitochondrial dysfunction, and cell senescence. Burgeoning evidence implies that loss of SIRT1 expression stimulates pro-atherogenic changes and orchestrates deleterious effect [20–22].

miR34a-5p is shown to be functional in cardiovascular biology as well as regulating circadian period gene. Based on our in-silico studies, we hypothesized miR34a-5p to be facilitating chronodisruption mediated pro-atherogenic manifestations. Herein, we decipher circadian relation of miR34a-5p, regulation of CLOCK/SIRT1 expression and subsequent pro-atherogenic changes in human endothelial cells and C57BL/6J mice subjected to photoperiodic manipulations induced chronodisruption.

## Materials and methods

### Chemicals and reagents

Cell culture products like HiEndo XL Endothelial Cell expansion medium, EnVzyme, bovine serum albumin (BSA) and antibiotic-antimycotic solution, Gelatin were purchased from Hi-media laboratories (Mumbai, India). iScript cDNA synthesis kit (1708891) and iTaq Universal SYBR Green SMX 100 (1725122) master mix were procured from BioRad (CA, USA). TRIzol was procured from Invitrogen (CA, USA). Antibodies Clock (PA1-520), β-actin (PA1-183), secondary anti-rabbit (32460) and ActinRed 555 Ready Probes reagent (R37112) were procured from Invitrogen (USA) and SIRT1 (SC-74465) and secondary anti-mouse m-IgGk (SC-516102) were purchased from Santa Cruz (Dallas, USA). miRNeasy kit (217004) and miRScript II RT kit (218161) were procured from Qiagen. RNA-later stabilizing solution was purchased from Ambion Inc. (USA). Haematoxylin, eosin and were purchased from Sigma Aldrich (St. Louis, MO, USA). Methanol, dimethyl sulphoxide (DMSO), 3-(4,5-dimethylthiazol-2-yl)-2,5-diphenyl tetrazolium bromide (MTT) were purchased from Sisco research laboratory pvt. ltd. (Mumbai, India).

### Computational studies

miRNA targets for CLOCK gene in human and mice were investigated using computational prediction algorithms (miRDB V 6.0 and TargetScan V 7.0) software. Potential miRNAs were further screened for functionality test (miRDB/ PubMed/ preliminary data from lab). Based on the results, miR34a-5p was identified as a potential candidate and subjected to gene target prediction using miRDB and TargetScan Version 7.0. PubMed evaluation for 899 genes targets of miR34a-5p was conducted and the percentage genes relevant to cardiovascular diseases were identified.

### Animal studies and experimental protocol

Male C57BL/6J mice, 6–8 weeks of age were procured from ACTREC Mumbai. Animals were maintained as per CPCSEA standard guidelines (23 ± 2°C, LD 12:12, laboratory chow and water ad libitum) for a week-long acclimatization before initiating the experiment. Experimental protocol was approved by Institutional Animal Ethical Committee (IAEC) (Approval no. MSU-Z/IAEC03/01-2019) and experiments were conducted in CPCSEA approved animal house facility of Department of Zoology, The Maharaja Sayajirao University of Baroda, Vadodara, India (827/GO/Re/S/04/CPC-SEA). All animal experimentations were done abiding to the ARRIVE guidelines.

Mice were randomly divided into 3 groups viz. (i) time point group (TP; LD: 12:12; n = 25); untreated mice sacrificed at ZT 0, 6, 12, 18, 24 (n = 5/time point). (ii) Control group (C; LD: 12:12; n = 6) and (iii) photoperiod induced chronodisruption group (CD; phase advance/phase delay photoperiodic schedule for 18 weeks; n = 6) [23, 24]. Briefly, mice were subjected to 7:00 to 19:00 h light/19:00 to 7:00 h dark period. The photoperiodic regimen was altered by giving 11:00 to 23:00 h dark/23:00 to 11:00 h light period, resulting in a phase advance of 8 hours (lights off at ZT4) and back to (7:00 to 19:00 hours light/19:00 to 7:00 hours dark period) schedule, resulting in a phase delay of 8 hours on 2^nd^ and 5^th^ day, respectively (S4 Fig). Experiment was terminated at end of 18^th^ week in morning 7:00 h (ZT0). Animals of group I were sacrificed at different time points (ZT 0, 6, 12, 18, 24) and animals of group II and III were sacrificed at ZT0. Serum was collected by retro orbital-sinus puncture under mild isoflurane anesthesia, Whole blood was centrifuged (at 4°C and 3000 rpm for 10 min) for serum collection. Animals were euthanized through cervical dislocation, and thoracic aortae were stored in 4% PFA (for histological evaluation, staining), in RNAlater (for RNA related studies) and in -80 refrigerator (for protein isolation).

### Serum biochemical parameters

Serum lipid profile (TL, TC, TG, LDL, VLDL, CHL/HDL, LDL/HDL ratio) were estimated using commercially available kits (Reckon Diagnostic kits, Vadodara, Gujarat, India). Atherogenic index of plasma (AIP) was calculated using the formula *log*_*10*_ *(TG/HDL-C)* and Cardiac risk Ratio (CRR) was calculated by *TC/HDL- C* [25].

### Histopathological analysis

Thoracic aorta (n = 6/group) were fixed in PFA (4%, pH 7.2) were dehydrated and embedded in paraffin wax blocks. Serial sections of 5 μm were cut on microtome and stained with hematoxylin and eosin (H&E), observed, and photographed (Nikon eclipse Ti2-E, Tokyo, Japan). Intima-media thickness (IMT) that was measured using FiJi software (ImageJ, NIH, Bethesda, USA).

Elastin autofluorescence was observed and photographed using FLoid imaging station (Thermo Fisher Scientific, USA). Derangement of elastin microstructure was evaluated as elastin fragmentation, (observed as niches and overall arrangement) and compared with control tissue.

Picrosirius red staining was used to study the collagen content in aorta wherein, sections were stained with 0.1% direct red 80 (Sigma Aldrich, USA) in saturated aqueous solution of picric acid for 1 h at RT and observed under Nikon eclipse Ti2-E (Tokyo, Japan). Images were captured, and quantitative analysis of collagen and elastin content was done using FiJi software (ImageJ, NIH, Bethesda, USA). Aortic stiffness was calculated as collagen: elastin ratio.

### Immunohistochemistry

5 μm thick sections of thoracic aortas were processed for immunohistochemistry. Sections were deparaffinized in xylene and rehydrated in graded series of ethanol. Sodium citrate buffer was used for antigen retrieval for 20 min at 95°C. Endogenous peroxidases were masked with 3% hydrogen peroxide for 20 min in dark. Further, sections were blocked with 1% BSA and incubated overnight in primary antibody clock (1:250; Invitrogen, USA) at 4°C in humidified chamber. Later, sections were incubated with horseradish peroxidase (HRP) conjugated secondary antibody (Dako, Agilent, USA) for 1 h at RT and DAB substrate (Dako, Agilent, USA) and counter-stained with hematoxylin and photographed using Nikon eclipse Ti2-E (Tokyo, Japan). Quantification of positively stained regions was carried out using Fiji software (ImageJ, NIH, Bethesda, USA).

### Cell culture

Human umbilical vein endothelial cells (HUVEC) were procured from HiMedia (Mumbai, India) and cultured in HiEndo XL Endothelial Cell expansion medium in CO_2_ Incubator (Thermo scientific, forma series II 3110, USA) at 37°C and 5% CO_2._ Cells were passaged at 80% confluency, using EnVzyme wherein cells of passage No. 2–7 were used in all the experiment. Circadian rhythm of cells was synchronized by giving serum shock (50% FBS for 2h) before experimentation [26, 27].

### Cytotoxicity assay

HUVEC were seeded in gelatin coated 96 well plate with HiEndoXL media and treated with different concentrations of LPS (2.5–20 μg/mL), dosed for 24h. Freshly prepared MTT (3-(4,5-dimethylthiazol-2-yl)-2,5-diphenyltetrazolium bromide, 5mg/ml in HiEndoXL media) was added in each well and incubated for 4h as per [28]. Resultant formazan crystals were dissolved in DMSO, and absorbance was measured at 570 nm using Synergy HTX multimode reader (Germany).

### Immunocytochemistry

HUVEC were cultured on gelatin coated coverslips. Circadian clock of the cells was synchronized by subjecting them to 50% FBS for 2h. Further, cells were dosed with 20 μM LPS for 24h in HiEndoXL media. Briefly, cells were washed with 1x PBS and fixed with 4% PFA for 15 min. Permeabilization was done with 0.1% Triton-x 100 in TBS followed by blocking with 3% BSA-PBS for 30 min. Cells were probed with CLOCK primary antibody (1:150; Invitrogen, USA) overnight at 4°C in humidified chamber. Later, cells were washed thrice with 1x PBS and incubated with Alexa Fluor-488 anti-rabbit secondary antibody (Invitrogen, Thermo Scientific, USA) along with ActinRed 555 Ready probes reagent (Invitrogen, Thermo Scientific, USA) for 1.30 hours. Cells were again washed thrice and mounted using Fluoroshield with DAPI (Sigma-Aldrich, USA). Imaging was done on Nikon eclipse Ti2-E (Tokyo, Japan).

### Gene expression

Total RNA was isolated was isolated from cells/tissue using TRIzol reagent and miRNA using miRNeasy kit. cDNA was synthesized using iScript cDNA synthesis kit (Bio-Rad, USA) and miScript II RT kit (Qiagen, Germany) for mRNA and miRNA respectively. Expression levels of mRNA and miRNA were determined using qPCR (Quantstudio 12K Flex RealTime PCR system, ThermoFisher Scientific, USA) using specific primer sequences (S2 Table) and SYBR Select master mix. The data were normalized using internal control 18S and 5S for mRNA and miRNA respectively [29–31]. Analysis was done with 2−ΔΔCT method.

### Immunoblot analysis

Thoracic aorta and HUVEC cells were homogenized using ice cold 1X RIPA buffer with protease inhibitory cocktail (Sigma Aldrich, USA). Total protein was quantified by Bradford assay. SDS PAGE (10% polyacrylamide gel) was performed with equal amount of protein loading, that were transferred to PVDF membrane (Bio-Rad, USA). Primary antibodies for Sirt1, Clock, BMAL1 (1:1000) were added followed by secondary anti-rabbit horseradish peroxidase antibody (1:1500)/ anti-mouse horseradish peroxidase antibody (1:5000). Blots were stripped with stripping buffer and re-probed with housekeeping beta-actin antibody (1:1000). Blots were developed using ECL reagent (Bio-Rad, CA, USA). Development was done on X-ray films.

### Luciferase reporter gene assay

Luciferase reporter gene assay was performed using Secrete-PairTM dual luminescence assay kit (Gene Copoeia, Rockville,MD, USA) as previously reported [32]. HUVEC were transfected either with 100 nM miScript hsa-miR-34a-5p mirVana mimic or negative control (Ambion, Waltham, MA, USA) and with either a plasmid containing a miTarget™ 3′ UTR CLOCK luciferase reporter (pEZX-MT05-CLOCK) or mutated miTarget™ 3′ UTR (pEZX-MT05-Mutant) (Genecopoeia, Rockville, MD, USA) for 48h. Gluc/SEAP assay was performed with 100 μL of luminescent and 10 μL of the collected supernatant from each group with n = 3 technical replicates and read on luminometer (Molecular Devices Gemini XS Fluorescent Microplate Reader; Marshal Scientific, NH, USA). The luciferase units were measured as relative luciferase units (RLU) and normalized to total protein.

### DNA methylation assay

Genomic DNA was isolated from thoracic aorta of C57BL/6J mice using GeneJet Genomic DNA (gDNA) purification kit (ThermoFisher Scientific, USA) as per manufacturer’s protocol. To access the genomic methylation pattern, gDNA was deaminated using EpiJet Bisulfite conversion kit (ThermoFisher Scientific, USA) according to manufacturer’s protocol. The deaminated gDNA was further used as template for running methylation specific PCR (MSP Assay). CpG islands were determined in the promoter region of miR34a, several hundred base pairs upstream of precursor transcription start site and 5 sets of primers were used for methylated and unmethylated DNA region each as per [33]. Real-time PCR was performed with following conditions: 95°C for 10 minutes, followed by 40 cycles of 95°C for 30 seconds, 52°C for 30 seconds, and 60°C for 30 seconds. A reaction tube w/o template was used as negative control and all the samples were run with n = 3 technical replicates.

### Statistical analysis

All the values of the data are presented here are expressed as the mean ± standard deviation (SD). Non-parametric one sample t-test was used to compare the mean values of the two groups. One–way analysis of variance (ANOVA) was used for multiple group comparison. Statistical analysis was performed using Graph pad prism 8.1.1 software. Amplitude of mRNA/miRNA expression were analyzed using Circwave software v1.4. Curve of amplitude were calculated as per [23]. The criterion of statistical significance was *p*<0.05.

## Results

### Photoperiodic manipulations alter clock gene expression in thoracic aorta of C57BL/6J mice

**Fig 1:**
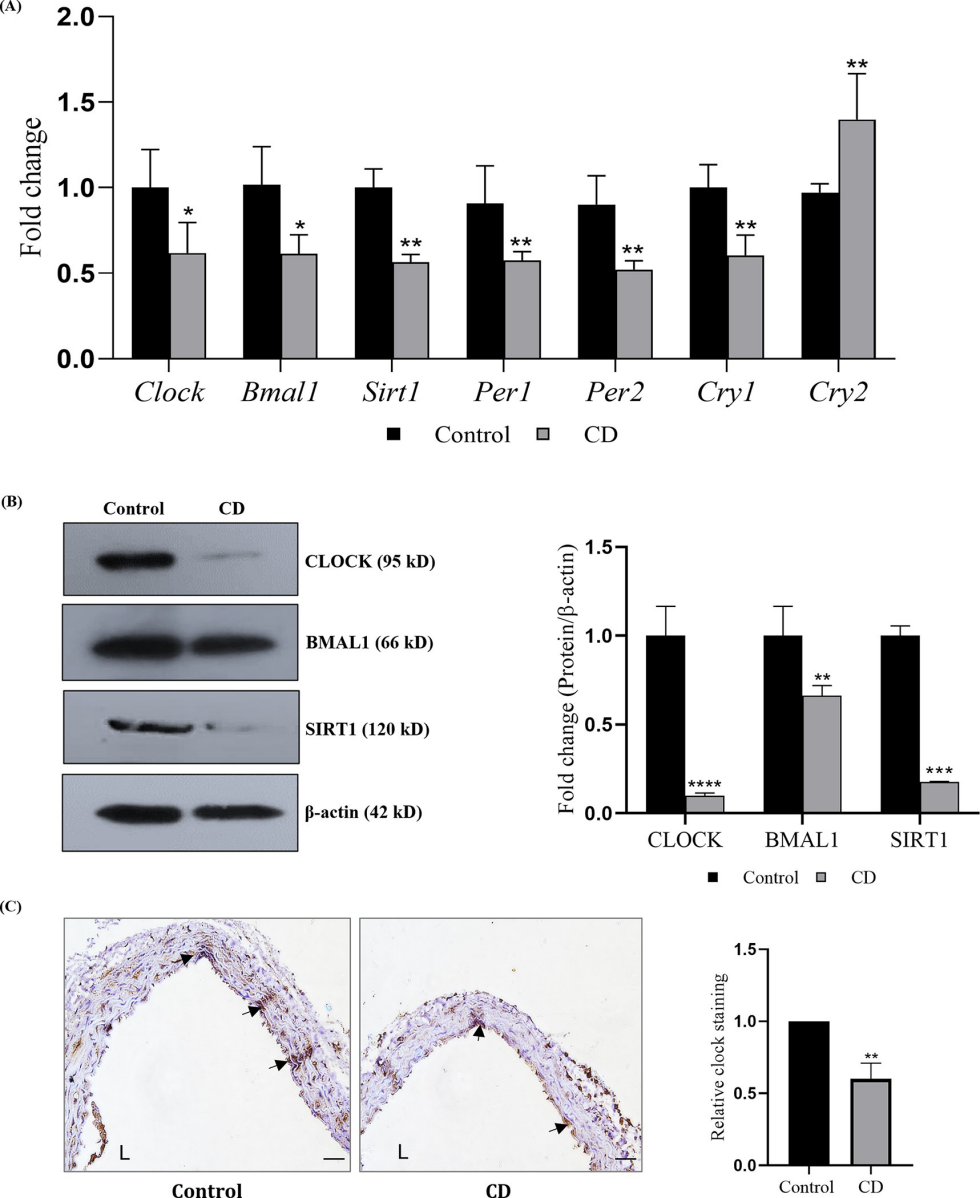
Photoperiodic manipulation induces chronodisruption in thoracic aorta of C57BL/6J mice. Mice (n = 6; 6–8 week) were subjected to phase advance/phase delay photoperiodic regime for 18 weeks and the experiment was terminated at ZT0 (7 a.m.) on the following day. (A) mRNA expression of circadian clock genes in the thoracic aortae (n = 6). (B) Protein expression of CLOCK, BMAL1 and SIRT1, normalized with housekeeping gene β-actin and their quantitative analysis (n = 4). (C) Immunohistochemistry of CLOCK gene expression in thoracic aorta (L indicates luminal area of the aorta and arrows indicate CLOCK staining) and its quantification (n = 3). Results are expressed as mean ± SD *p < 0.05, **p < 0.01 or ***p < 0.001 is when CD is compared to control group.

C57BL/6J mice were subjected to phase advance-phase delay photoperiodic regimen to induce CD for 18 weeks (S1 Fig), a model previously established in our lab [23]. Animals were sacrificed at ZT0, and thoracic aortae were collected. mRNA quantification showed that the levels of core clock genes (*Clock* and *Bmal1;* Fig 1A and 1B) and CCG (*Per1*, *Per2* and *Cry1;* Fig 1C and 1E) showed a significant decrement (*P*< 0.01) with reciprocal set of changes in *Cry2* in CD mice (Fig 1F). SIRT1, NAD^+^ dependent deacetylase was also found to be lowered in thoracic aorta of CD mice (Fig 1G). The immunoblots of CLOCK, BMAL1 and SIRT1 were in agreement with changes recorded at the mRNA level (Fig 1H and 1I). Moreover, immunohistochemical localization of CLOCK protein in thoracic aorta of CD mice also showed a lowered expression (Fig 1J and 1K). Lowered levels of core and CCG genes in the target tissue is indicative of photoperiod induced chronodisruption and the same was observed in thoracic aorta of CD mice.

### Chronodisruption elevates miR-34a expression by hypomethylation in its promoter region

**Fig 2:**
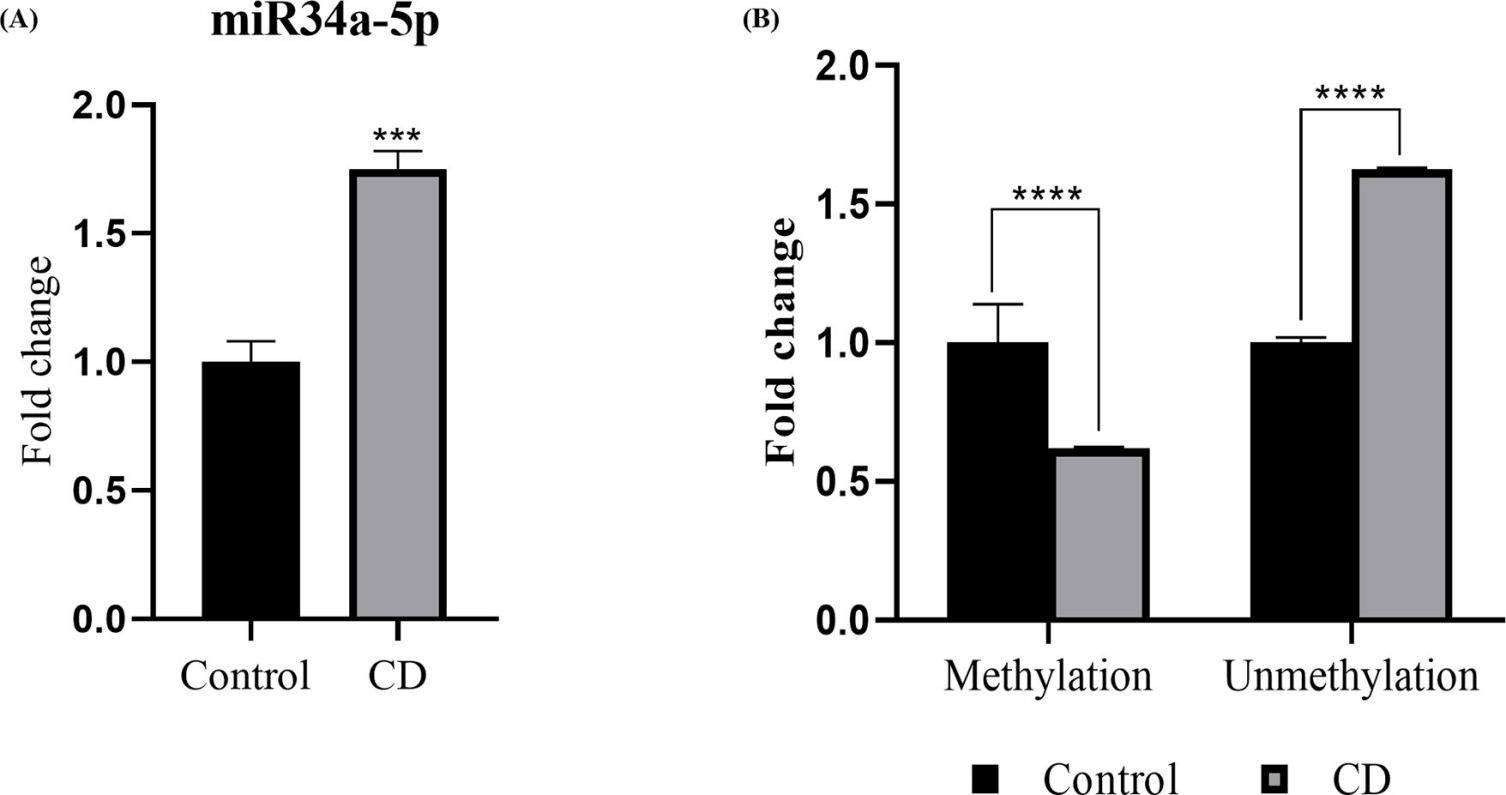
Chronodisruption elevates miR34a-5p levels by hypomethylation in its promoter region. (A) miR34a-5p expression in thoracic aorta of C57BL/6J mice. gDNA was isolated from the thoracic aorta and methylation pattern was evaluated in the promoter region of miR34a by MSP assay, results shown as quantitative (B) methylation and unmethylation in the promoter region (n = 4). Results are expressed as mean ± SD *p < 0.05, **p < 0.01 or ***p < 0.001 is when CD is compared to control group.

Higher levels of miR34a-5p have been reported in atherogenic milieu to sensitize crucial pathophysiological pathways viz. endothelial cell senescence, cholesterol efflux, macrophage polarization and atheromatous plaque development [34, 35]. In our study, we identified significantly elevated miR34a-5p levels in the thoracic aortae of CD mice (Fig 2A). In order to identify the cause, we assessed aberrant methylation in the CpG islands of gDNA has been closely associated with human disease development under influence of chronodisruption. Methylation specific PCR indicated significant hypomethylation in the CpG island of the promoter region, about 300bp upstream of the transcriptional start site of miR34a-5p in case of CD aortae (Fig 2B and 2C). These findings indicate that chronodisruption orchestrates epigenetic modifications involving promoter hypomethylation resulting in elevated miR-34a-5p expression.

### miR34a-5p exhibits complementarity to the seed sequence in 3’UTR of clock gene

**Fig 3:**
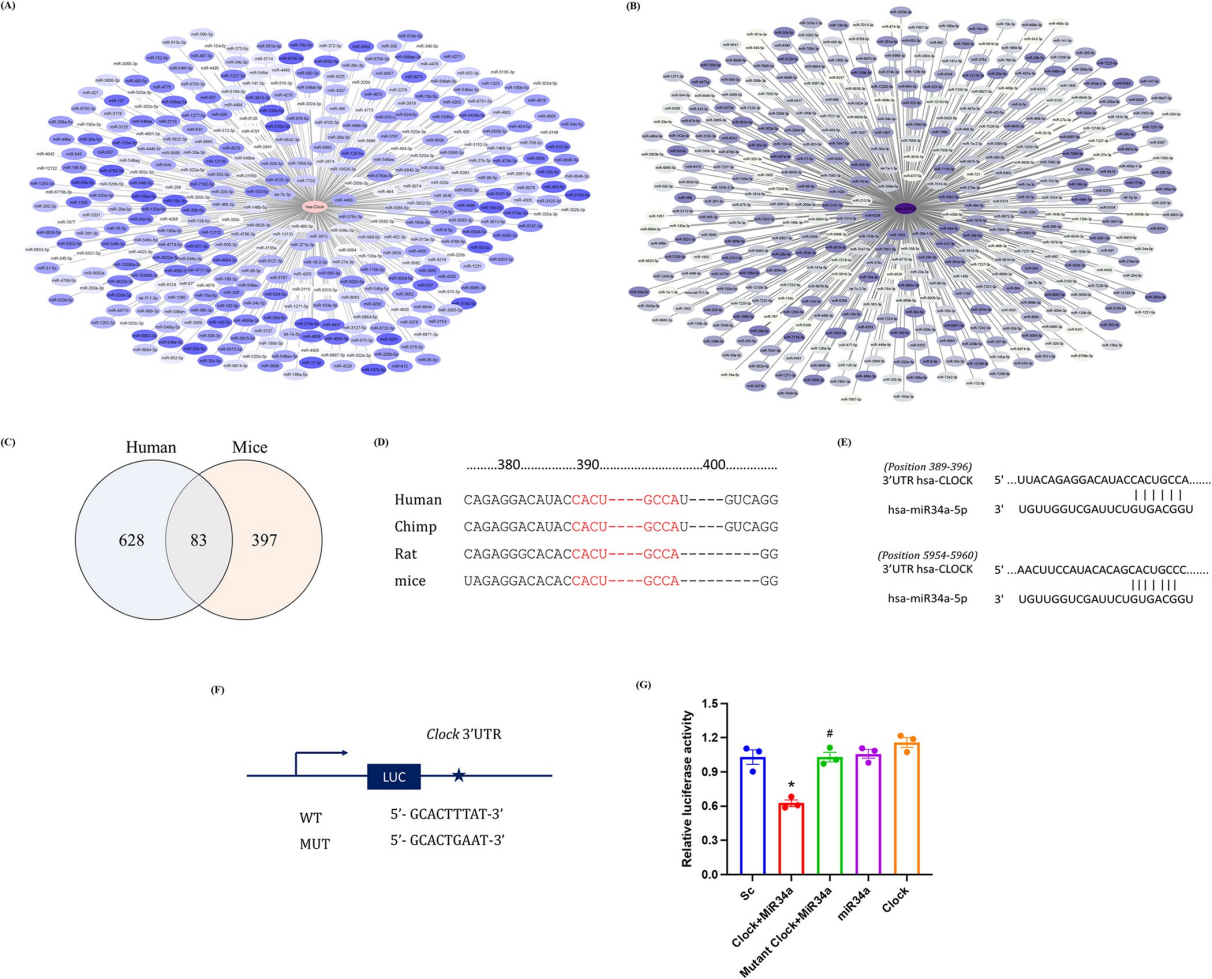
miR34a-5p potentially binds to 3’UTR of CLOCK. Computational analysis of miRNA-mRNA target prediction was assessed using miRDB V 6.0 and TargetScan V 7.0. Interacting network of miRNAs with complementary seed sequence to (A) *hsa-Clock* (gradient from white to blue indicates increasing hit Score) and (B) *mmu-Clock* (gradient from white to lilac indicates increasing hit Score) was constructed using Cytoscape. (C) Venn diagram shows number of unique and common miRNAs interacting with 3’UTR of *Clock* in human and mice. (D) Conserved miR34a-5p binding sites on 3’UTR of gene *Clock* in different vertebrates (depicted in red). (E) Schematic representation of 3’UTR of *hsa-Clock* exhibiting two seed sequences complementing miR34a-5p. (F) Schematic construct of vector with wild type or mutated binding sites on 3’UTR of *Clock* interacting to miR34a-5p. (G) HUVEC cells were transfected with WT and mutated 3’UTR *Clock* gene assessed for luciferase activity with/without miR34a-5p mimics (n = 3). Results are expressed as mean ± SD *p < 0.05, **p < 0.01 or ***p < 0.001 Vs control group (SC). #*p* < 0.05, ##*p* < 0.01 or ###*p* < 0.001 vs WT clock + miR34a-5p mimic.

miR34a-5p is already reported to inhibit SIRT1 expression by impeding its 3’UTR activity [36–38]. To evaluate such plausible link between miR34a-5p elevation and subsequent CLOCK suppression, a computational approach was employed. In silico target prediction softwares miRDB V 6.0 and TargetScan V 7.0 were used to screen the set of miRNAs targeting the seed sequence in the 3’UTR of *Clock* gene. About 628, 397 and 163 miRNAs had potential complementarity to the seed location in 3’UTR of clock genes in human, mice and rat respectively (Fig 3A and 3B; S1 Table). Further evaluation suggested that seed sequence of miR-34a-5p, on the 3’UTR of clock gene, was conserved across several species over evolution (Fig 3C). The 3’UTR of human clock gene exhibits two seed sequences at 389 and 5954 nt (Fig 3D), whereas mice and rat exhibit one each at 369 and 363 nt respectively (S1 Table).

### Luciferase reporter gene assay confirms miR34a-5p binding to 3’UTR of CLOCK gene

To confirm the *in-silico* data and miR-34a-5p inhibitory activity towards the CLOCK expression, luciferase reporter gene assay was performed in primary endothelial cells. HUVEC cells were co-transfected with miR-34a-5p mimic and plasmid containing a miTarget™ 3′ UTR CLOCK luciferase reporter (Fig 3E). The luciferase activity of the group co-transfected with miR-34a-5p mimic was significantly reduced as compared to those of HUVEC transfected with a scramble miR or with a reporter clone, used as control for off-target effects. Further validation was obtained by co-transfecting miR34a-5p mimic with a mutant reporter clone. Alteration in the seed sequence brought by point mutation (mutant reporter clone) failed to provide Watson-Crick complementarity to miR-34a-5p, allowing full expression of reporter gene and luciferase activity comparable to the scramble control (Fig 3F). These experiments suggest that miR-34a-5p inhibits CLOCK expression by impeding its 3’UTR activity.

### Elevated miR-34a lowers CLOCK and SIRT1 expression in HUVEC

**Fig 4:**
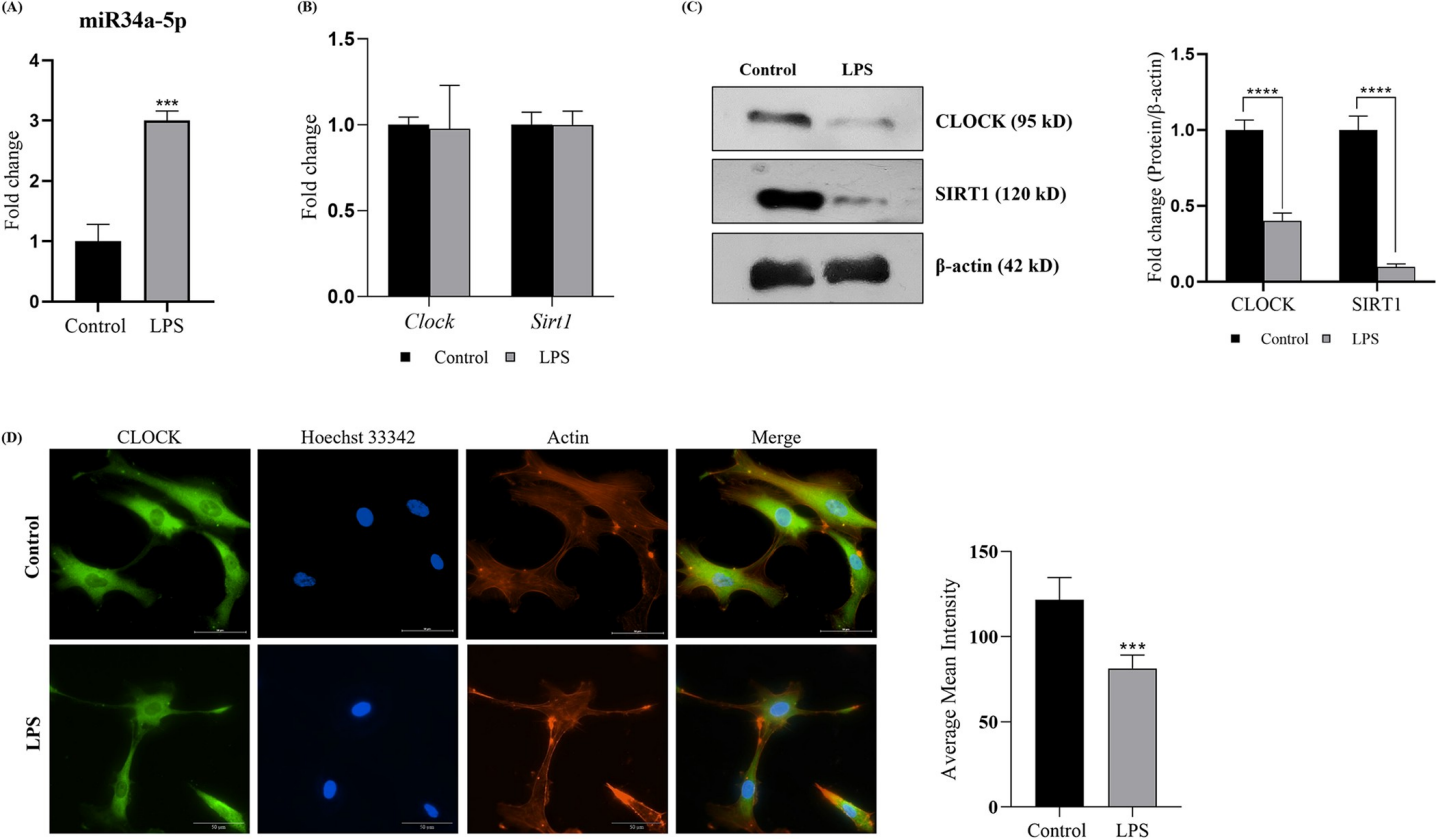
Elevated miR34a-5p lowers CLOCK protein expression in HUVEC cells. Circadian rhythm of HUVEC cells was synchronized with serum shock (50% FBS) for 2h for all the experimentations. Cells were dosed with 20 μg/mL of LPS for miR34a-5p upregulation and the experiment was terminated at 24h. (A) Levels of miR34a-5p in HUVEC (n = 3). (B) mRNA expression of *Clock* and *Sirt1* (n = 3). (C) Immunoblot analysis of CLOCK and SIRT1 proteins normalized with β-actin (housekeeping gene, n = 3) and its quantification. (D) Immunocytochemical analysis of CLOCK expression in HUVEC cells (n = 3; scale: 50μm) and its quantitative analysis. Results are expressed as mean ± SD *p < 0.05, **p < 0.01 or ***p < 0.001 is when LPS treatment is compared to control group.

In order to assess the expressional correlation between miR34a-5p, CLOCK and SIRT1 in an *in vitro* condition, we utilized serum synchronized HUVEC (ssHUVEC). Lipopolysaccharide (LPS) is reported to elevate miR34a-5p titers in HUVEC [39] along with orchestrating pro-atherogenic manifestations [40] and thus the same was used for miRNA upregulation. LPS toxicity was assessed by performing cell viability assay (MTT assay) with different doses ranging between 2.5–20 μg/mL. ~80% cells were found to be viable at the highest dose (20 μg/mL; S4 Fig) along with Significant increment in miR34a-5p expression following 24h of LPS treatment (Fig 4A). The mRNA levels of *Clock* and *Sirt1* genes were comparable to control (Fig 4B and 4C). However, it is interesting to note that the protein expression of CLOCK and SIRT1 were found to be significantly lowered (Fig 4D and 4E). The same was also observed in the immunocytochemical localization of clock gene in LPS treated/untreated synchronized HUVEC cells (Fig 4F). These data imply towards miR34a-5p mediated inhibition of CLOCK and SIRT1 expression.

### miR34a-5p exhibited antagonistic circadian oscillation to Clock and Sirt1 expression in HUVECs

**Fig 5:**
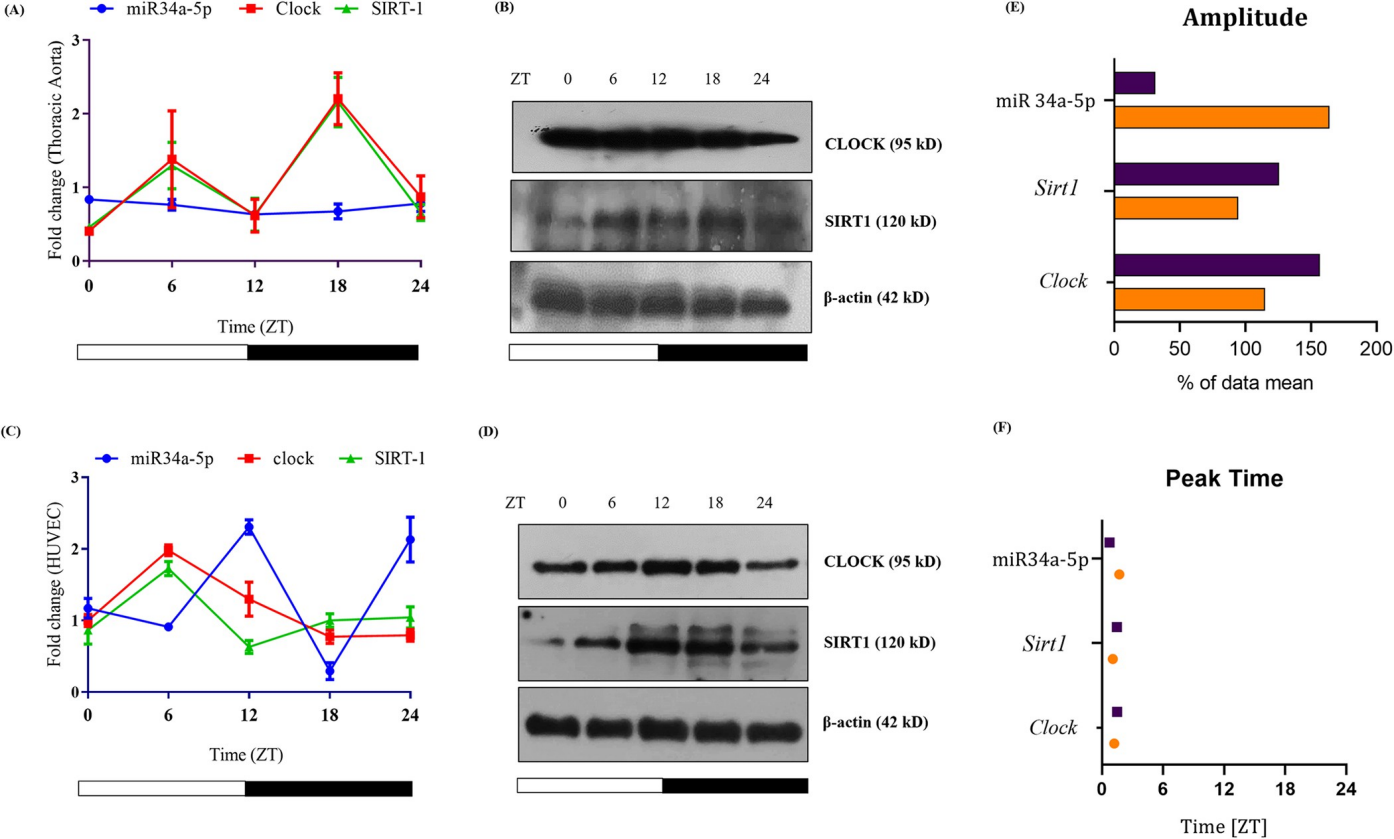
Circadian pattern miR34a-5p corresponding to its targets CLOCK and SIRT1. C57BL/6J mice (12:12 LD regimen) were sacrificed at 5 different time points (ZT 0, 6, 12, 18 and 24) and thoracic aortae were collected. (A) Circadian pattern of miR34a-5p and corresponding mRNA expression of *Clock* and *Sirt1* (B) protein expression of CLOCK and SIRT1, normalized with β-actin (housekeeping gene). HUVEC cells were serum synchronized with 50% FBS for 2h. Cells were harvested at five different time points corresponding to *in vivo* experiments, (ZT 0, 6, 12, 18 and 24). (C) Circadian pattern of miR34a-5p and corresponding mRNA expression of *Clock* and *Sirt1* (D) Protein expression of CLOCK and SIRT1 normalized with β-actin (housekeeping gene). Assessment of (E) Amplitude and (F) peak time of miR34a-5p, *Clock* and *Sirt1* were done with Circwave Software. Marked thoracic aorta (purple) and HUVEC cells (Orange).

Circadian rhythm of miR-34a-5p were studied with regards to *Clock* and *Sirt1* levels from thoracic aorta of C57BL6/J mice at five different time points within 24h (ZT 0, 6, 12, 18, 24). Both *clock* and S*irt1* mRNAs showed a synchronous pattern with a peak at ZT 6 and ZT 18. On the contrary, miR34a-5p expression showed a linear pattern that was not in synchrony with the observed peaks of *Clock* and S*irt1* mRNA and protein in the thoracic aorta (Fig 5A and 5B). miRNAs are known to oscillate independently in different tissues and cell types [miR25, miR34] [7, 13]. We assessed the circadian rhythm of miR34a-5p by performing a time point study (ZT 0, 6, 12, 18, 24 h) in HUVEC to mimic the response of tunica intima of thoracic aorta. Peak was detected at ZT6 for both CLOCK and SIRT1 mRNAs, whereas miR34a-5p showed a circadian pattern with a prominent ebb at ZT6 and ZT18 and peaks at ZT12 and ZT24 (Fig 5C). These findings were further validated with immunoblot and Circwave analysis of CLOCK, SIRT1 and miR-34a-5p (Fig 5C and 5D). Amplitude of miR34a-5p was observed to be higher in HUVEC as compared to that of aorta. However, amplitudes of *clock* and S*irt1* were marginally higher in aorta (Fig 5E). Peak timings of the genes and miRNA calculated as Center of Gravity (COG) were comparable in both HUVEC and thoracic aorta (Fig 5F).

### Chronodisruption induces pro-atherogenic manifestation in thoracic aorta of C57BL/6J mice

**Fig 6:**
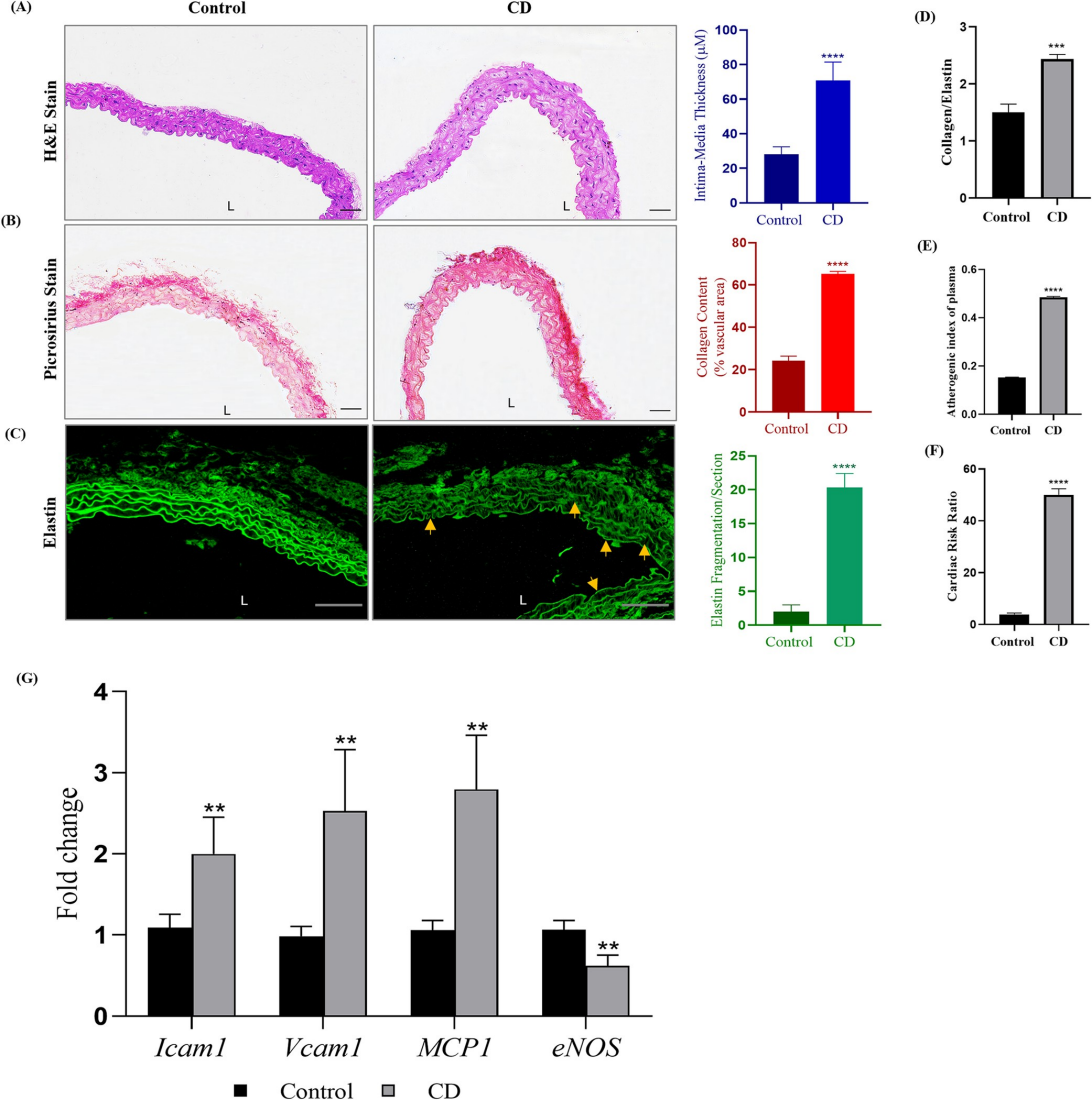
Chronodisruption induced pro-atherogenic manifestation in thoracic aorta of C57BL/6J mice. Mice (n = 6; 6–8 week) were subjected to phase advance/phase delay photoperiodic regime for 18 weeks. Histomorphological assessment was done as (A) H&E staining and derived statistical quantification of intimal-media thickening of the aortae (B) Picrosirius Red staining and its quantification (C) Elastin autofluorescence and quantification of elastin fragmentation and (D) Collagen to elastin ratio of thoracic aorta indicative of arterial stiffening. Serum lipid profiles of the mice were analyzed and calculated as (E) Atherogenic index of plasma (AIP) and (F) Cardiac risk ratio (CRR). (G) mRNA expression of key atherogenic genes. Results are expressed as mean ± SD *p < 0.05, **p < 0.01 or ***p < 0.001 is when CD is compared to control group.

Chronodisruption mediated elevation of miR34a-5p lowered SIRT1 titers in the thoracic aortae of CD mice. Burgeoning evidence report lowered SIRT1 levels to be associated to pro-atherogenic manifestations in different model system [41]. Thus, we decided to evaluate pro-atherogenic manifestations in the thoracic aorta of C57BL/6J mice. CD mice showed non-significant changes in body weight, body circumference, food and water intake (S2A–S2E Fig). Serum lipid profiles of CD mice showed significant increment in circulating triglycerides, total cholesterol and LDL-c and decreased levels of HDL-c (S3 Fig) resulting in significantly higher Atherogenic Index of Plasma (AIP) and Cardiac Risk Ratio (CRR) (Fig 6E and 6F).

Gross morphological evaluation of H&E-stained thoracic aortas of CD mice showed intimal derangement and higher intima: media thickening but, no evidence of atheromatous plaque formation (Fig 6A). Photomicrographs of elastin autofluorescence showed elastin fragmentation and fibrillar derangement (Fig 6C). Further, CD mice recorded significantly higher collagen content (picrosirius red stain) and a higher collagen/elastin ratio (Fig 6B and 6D). The altered collagen-elastin content in CD mice further suggested pro-atherogenic changes. Overall, the observed microscopic changes in thoracic aorta of CD mice imply towards early proatherogenic changes manifested by the photoperiodic manipulation induced chronodisruption.

mRNA levels of pro-inflammatory genes viz. intercellular adhesion molecule 1 (*Icam1*) and vascular endothelial cell adhesion molecule-1 (*Vcam1*) along with monocyte chemo-attractant protein-1 (*MCP-1*) were significantly upregulated in aorta of CD mice. On the contrary, mRNA levels of endothelial nitric oxide synthase (*eNOS*) were significantly lower in aortae of CD mice than control (Fig 6G). Overall, the data provides confirmatory evidence on CD-induced proatherogenic manifestations in thoracic aorta and are in agreement with our previous studies (24).

## Discussion

Circadian misalignment has increasingly gained prominence as one of the key causative factors contributing to the onset and progression of cardiovascular disorders including atherosclerosis [40]. The present study is aimed at investigating the association between circadian alterations and subsequent pro-atherogenic manifestations in thoracic aorta of C57BL/6J mice subjected to constant cyclic phase advanced/phase delay photoperiodic regimen [23]. Our study had revealed that histopathological changes and altered Clock gene expression in CD mice were associated with an increased expression of miR34a-5p. The same was previously implicated in endothelial cells dysfunction and increased atherogenesis [42].

In recent years, miRNAs have emerged as key regulators of fine-tuned gene expression in several physiological and pathological processes [43–45]. miRNAs are cell-type enriched and operate by regulating gene network dynamically and/or transiently (i.e., feed forward loop, feedback loop), along with steady state gene regulation (translational/transcriptional control). miR34a is shown to have several pathological implications in endothelial cells. To gauge the status of miR34a-5p in the chronodisruptive conditions, we developed a CD model by subjecting mice to a constant phase advance and phase delay photoperiodic regimen as reported in our previous study [23]. Wherein, we recorded elevated expression of miR34a-5p in the thoracic aortae of CD mice. Chronodisruption is often reported to cause aberrant methylations in the genomic DNA. miR34a is an intergenic miRNA with unique identifiable promoter region and is transcribed independently [33]. Moreover, promoter region of miR34a tends to undergo epigenetic changes as reported in several cancer cell lines [46–48]. So, we assessed epigenetic modifications in the CpG island in the promoter region with MSP assay to record a significant hypomethylation in the aortae of CD mice facilitating the elevated expression of miR34a-5p.

Interestingly, an inverse expressional co-relation was observed between miR34a-5p and CLOCK and SIRT1 genes in CD aortae. miR34a-5p is known to regulate SIRT1 expression by inhibiting its 3’UTR activity [36–38]. Similarly, several miRNAs are known to regulate clock gene expression to alter circadian rhythms viz. miR-25 –*Per2*, miR-17-5p –*Clock*, miR34a –*Per2*, miR-191 –*Bmal1* [7, 10, 11, 13]. So, we did computational algorithmic analysis of miR34a-5p –*Clock* association (miRDB and Target Scan) that showed 3’UTR of clock gene exhibits complementary seed sequence to miR34a-5p which was conserved across species. 3’UTR of hsa-CLOCK exhibits 2 seed sequences whereas mmu-CLOCK and rno-CLOCK exhibits one seed sequence each (S1 Table). Luciferase reporter gene assay in HUVEC cells had confirmed the *in-silico* findings of miR34a-5p regulating *Clock* expression by binding to its 3’UTR. Overall, hypomethylation in CD conditions had led to elevated miR34a-5p titers accounting for a lowered *Clock* gene expression.

The regulatory control of miRNAs on circadian genes implies a possibility of the said miRNAs exhibiting oscillations in synchrony with the circadian genes. Circadian rhythmic expression of miR34a is reported in transformed cell lines [13]. Also, modulation of certain miRNAs is known to alter the phase and amplitude of circadian clock genes [7]. These two sets of studies underline the fact that miRNAs too have a circadian cycle synchronous to the clock genes. So, we did a time point based study that first-hand reports the cyclic expression of miR34a-5p in ssHUVEC cells. Contrary to this observation, an oscillatory pattern of miR34a-5p was not seen in thoracic aortae. Dampened oscillations of miR34a-5p recorded are possibly attributable to a complex histo-architecture of the aorta comprising of diverse cell types in which, the tunica intima contributes to a small subset of the total mass. Larger oscillatory amplitude of SIRT1 and CLOCK, unlike miR34a-5p, in the thoracic aortae suggest a fine-tuning role of miR34a-5p in circadian clock regulation [10, 49]. Moreover, the oscillations of miR34a-5p were antagonistic to that of *Clock/ Sirt1* in HUVEC with its peak timings corresponding to the ebb of the genes, implying towards miRNA-based target-gene regulation. We further checked circadian pattern of genes under elevated mir34a-5p levels, as it would be under CD conditions. ssHUVEC treated with LPS, induced upregulation of miR34a-5p that had accounted for a complete loss of cyclicity of CLOCK and SIRT1 expressions. SIRT1 deacetylase and CLOCK inhibition would disable operation of positive arm of circadian clock and transcription of CCGs (*Per1*, *Per2*, *Cry1*, *Cry2*) along with failed *Per2* deacetylation that would complete the feedback loop by inhibiting CCG transcription [1, 2]. The observed higher levels of miR34a-5p had accounted for imperative perturbations in circadian clock expression in HUVEC and therefore miR34a-5p can be stated as a chronodisruptive miRNA.

Physiologically SIRT1 is positioned at a chrono-metabolic juncture and is reported to be functional at several stages of initiation and development of atherosclerosis. Elevated levels of miR34a-5p have been reported to inactivate 3’UTR function of Sirt1 [37, 42, 50]. These reports corroborate our findings of higher levels of miR34a-5p and concomitant decrement in SIRT1 as a one of the triggering factors for proatherogenic manifestations evidenced in the thoracic aortae of CD mice.

**Fig 7:**
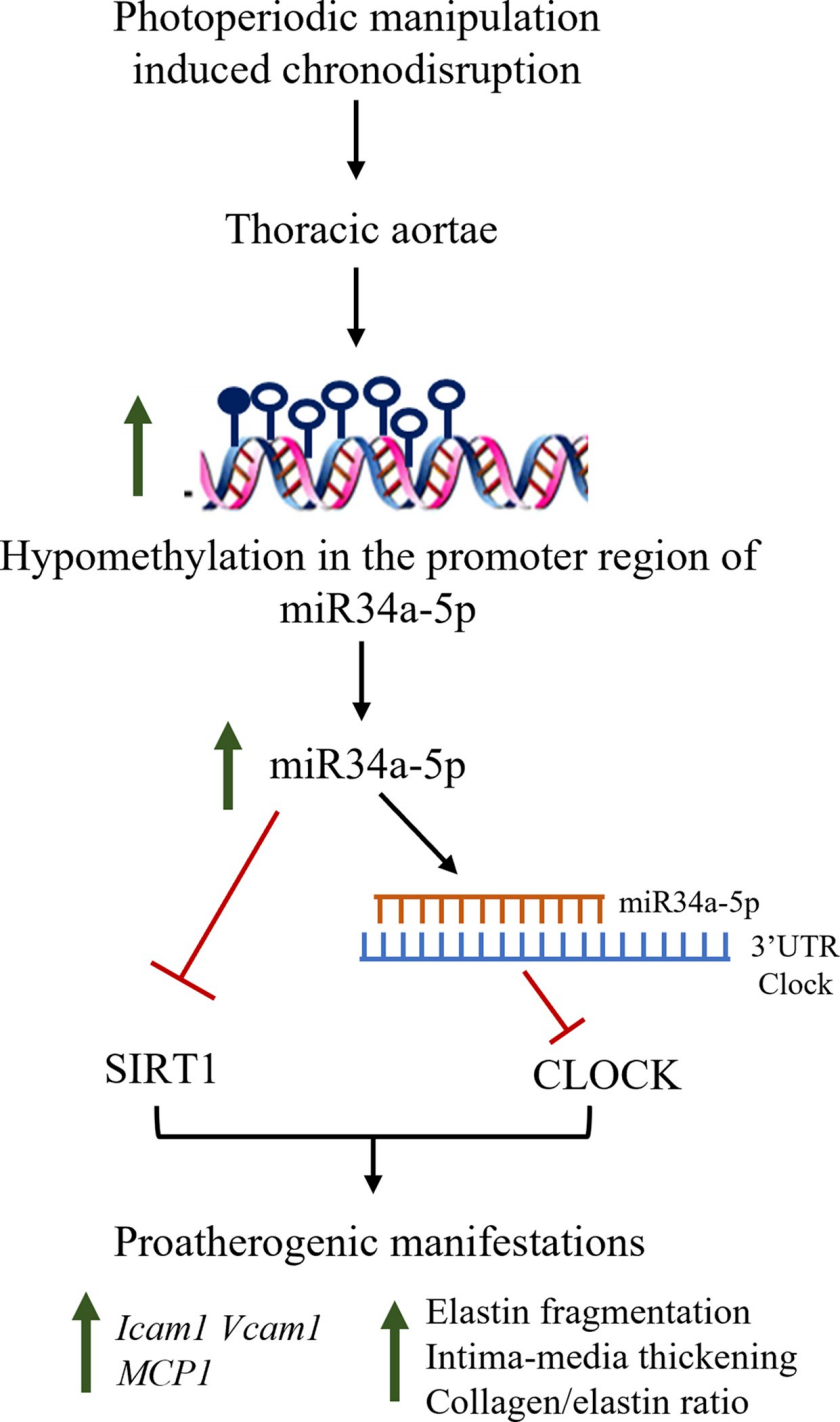
Summary diagram of the study depicts that photoperiodic manipulation induced chronodisruption causes hypomethylation in the promoter region of miR34a-5p resulting in its elevation. miR34a-5p impedes CLOCK expression by inhibiting its 3’ UTR activity, and SIRT1 expression. Physiologically, miR34a-5p oscillates in antagonistic circadian pattern to CLOCK and SIRT1. Cumulative alterations through miR34a-5p elevation manifest proatherogenic changes in the thoracic aortae of chronodisruptive mice.

In summary, this study establishes circadian correlation of miR34a-5p and mediated CLOCK regulation (Fig 7). It further proposes chronodisruption mediated elevation of miR34a-5p to potentiate pro-atherogenic manifestations by negatively regulating CLOCK and SIRT1 expressions.

## Supporting information

S1 Fig Schematic representation of photoperiodic regime employed for *in vivo* experiments. Control C57BL/6J mice were subjected to LD 12:12. Whereas CD mice were subject to 8h phase advance from day 2 and 8h phase delay from day 5 for 18 weeks.
(TIF)

S2 Fig (A) Biopsy of C57BL/6J mice. (B) Body weight (C) Body Circumference recorded at the end of experiment; (D) Food intake and (E) water intake recorded throughout the period of study in Control and CD mice.
(TIF)

S3 Fig (A-F) Serum lipid profile of control and CD mice. Results are expressed as mean ± SD *p < 0.05, **p < 0.01 or ***p < 0.001 for CD vs control group.
(TIF)

S4 Fig HUVEC cells were treated with ascending doses (2.5 to 20 μg/mL) of LPS and cytotoxicity (MTT) assay was performed after 24h. Data represents % viable cells at different concentrations.
(TIF)

S1 Table Total miRNAs that form watson-crick complementary base pairing to 3’UTR of clock gene. Also shown are the dock scores and seed regions of miR34a-5p and, promoter length of clock gene in vertebrates viz. mice, rat and human.
(DOCX)

S2 Table List of primers for human and mouse. (DOCX)

S1 Raw images Raw images of western blots. (PDF)

## References

[1] Asher G, Gatfield D, Stratmann M, Reinke H, Dibner C, Kreppel F, et al. SIRT1 regulates circadian clock gene expression through PER2 deacetylation. Cell. 2008;134(2):317–28. doi: 10.1016/j.cell.2008.06.050 18662546

[2] Belden WJ, Dunlap JC. SIRT1 is a circadian deacetylase for core clock components. Cell. 2008;134(2):212–4. doi: 10.1016/j.cell.2008.07.010 18662537 PMC3671948

[3] Chen R, Schirmer A, Lee Y, Lee H, Kumar V, Yoo S-H, et al. Rhythmic PER abundance defines a critical nodal point for negative feedback within the circadian clock mechanism. Molecular cell. 2009;36(3):417–30. doi: 10.1016/j.molcel.2009.10.012 19917250 PMC3625733

[4] Masri S, Cervantes M, Sassone-Corsi P. The circadian clock and cell cycle: interconnected biological circuits. Current opinion in cell biology. 2013;25(6):730–4. doi: 10.1016/j.ceb.2013.07.013 23969329 PMC4573394

[5] Bartman CM, Oyama Y, Brodsky K, Khailova L, Walker L, Koeppen M, et al. Intense light-elicited upregulation of miR-21 facilitates glycolysis and cardioprotection through Per2-dependent mechanisms. PloS one. 2017;12(4):e0176243. doi: 10.1371/journal.pone.0176243 28448534 PMC5407766

[6] Chen X, Rosbash M. MicroRNA-92a is a circadian modulator of neuronal excitability in Drosophila. Nature communications. 2017;8(1):1–11. 10.1038/ncomms14707 PMC5347142 28276426

[7] Park I, Kim D, Kim J, Jang S, Choi M, Choe HK, et al. microRNA-25 as a novel modulator of circadian Period2 gene oscillation. Experimental & molecular medicine. 2020;52(9):1614–26. doi: 10.1038/s12276-020-00496-5 32968200 PMC8080691

[8] Bartel DP. MicroRNAs: target recognition and regulatory functions. cell. 2009;136(2):215–33. doi: 10.1016/j.cell.2009.01.002 19167326 PMC3794896

[9] Friedman LM, Avraham KB. MicroRNAs and epigenetic regulation in the mammalian inner ear: implications for deafness. Mammalian genome. 2009;20(9):581–603. doi: 10.1007/s00335-009-9230-5 19876605

[10] Gao Q, Zhou L, Yang S-Y, Cao J-M. A novel role of microRNA 17-5p in the modulation of circadian rhythm. Scientific reports. 2016;6(1):1–12. 27440219 10.1038/srep30070 PMC4954982

[11] Chinnapaiyan S, Dutta RK, Devadoss D, Chand HS, Rahman I, Unwalla HJ. Role of Non-Coding RNAs in Lung Circadian Clock Related Diseases. International Journal of Molecular Sciences. 2020;21(8):3013. doi: 10.3390/ijms21083013 32344623 PMC7215637

[12] Han Y, Meng F, Venter J, Wu N, Wan Y, Standeford H, et al. miR-34a-dependent overexpression of Per1 decreases cholangiocarcinoma growth. Journal of hepatology. 2016;64(6):1295–304. doi: 10.1016/j.jhep.2016.02.024 26923637 PMC4874896

[13] Hasakova K, Reis R, Vician M, Zeman M, Herichova I. Expression of miR-34a-5p is up-regulated in human colorectal cancer and correlates with survival and clock gene PER2 expression. PLoS One. 2019;14(10):e0224396. doi: 10.1371/journal.pone.0224396 31658284 PMC6816564

[14] Reddy AB, Karp NA, Maywood ES, Sage EA, Deery M, O’Neill JS, et al. Circadian orchestration of the hepatic proteome. Current Biology. 2006;16(11):1107–15. doi: 10.1016/j.cub.2006.04.026 16753565

[15] Rudic RD, McNamara P, Reilly D, Grosser T, Curtis A-M, Price TS, et al. Bioinformatic analysis of circadian gene oscillation in mouse aorta. Circulation. 2005;112(17):2716–24. doi: 10.1161/CIRCULATIONAHA.105.568626 16230482

[16] Raitoharju E, Lyytikäinen L-P, Levula M, Oksala N, Mennander A, Tarkka M, et al. miR-21, miR-210, miR-34a, and miR-146a/b are up-regulated in human atherosclerotic plaques in the Tampere Vascular Study. Atherosclerosis. 2011;219(1):211–7. doi: 10.1016/j.atherosclerosis.2011.07.020 21820659

[17] Zhang L, Chen J, He Q, Chao Z, Li X, Chen M. MicroRNA‑217 is involved in the progression of atherosclerosis through regulating inflammatory responses by targeting sirtuin 1. Molecular Medicine Reports. 2019;20(4):3182–90. 31432137 10.3892/mmr.2019.10581 PMC6755250

[18] González-López P, Ares-Carral C, López-Pastor AR, Infante-Menéndez J, González Illaness T, Vega de Ceniga M, et al. Implication of miR-155-5p and miR-143-3p in the vascular insulin resistance and instability of human and experimental atherosclerotic plaque. International journal of molecular sciences. 2022;23(18):10253. doi: 10.3390/ijms231810253 36142173 PMC9499612

[19] Wu J, Liang W, Tian Y, Ma F, Huang W, Jia Y, et al. Inhibition of P53/miR‐34a improves diabetic endothelial dysfunction via activation of SIRT1. Journal of Cellular and Molecular Medicine. 2019;23(5):3538–48. doi: 10.1111/jcmm.14253 30793480 PMC6484332

[20] Tsai KL, Hung CH, Chan SH, Hsieh PL, Ou HC, Cheng YH, et al. Chlorogenic acid protects against oxLDL‐induced oxidative damage and mitochondrial dysfunction by modulating SIRT1 in endothelial cells. Molecular Nutrition & Food Research. 2018;62(11):1700928. 10.1002/mnfr.201700928 29656453

[21] Winnik S, Stein S, M Matter C. SIRT1-an anti-inflammatory pathway at the crossroads between metabolic disease and atherosclerosis. Current vascular pharmacology. 2012;10(6):693–6. doi: 10.2174/157016112803520756 23259556

[22] Bai B, Vanhoutte PM, Wang Y. Loss-of-SIRT1 function during vascular ageing: hyperphosphorylation mediated by cyclin-dependent kinase 5. Trends in cardiovascular medicine. 2014;24(2):81–4. doi: 10.1016/j.tcm.2013.07.001 23968571

[23] Joshi A, Upadhyay KK, Vohra A, Shirsath K, Devkar R. Melatonin induces Nrf2‐HO‐1 reprogramming and corrections in hepatic core clock oscillations in non‐alcoholic fatty liver disease. The FASEB Journal. 2021;35(9):e21803. doi: 10.1096/fj.202002556RRR 34365685

[24] Shirsath K, Joshi A, Vohra A, Devkar R. Chronic photoperiodic manipulation induced chronodisruption upregulates HSP60 during early pro-atherogenic remodeling in thoracic aorta of C57BL/6J mice. The Journal of Basic and Applied Zoology. 2021;82(1):1–10.

[25] Cheraghi M, Shahsavari G, Maleki A, Ahmadvand H. Paraoxonase 1 activity, lipid profile, and atherogenic indexes status in coronary heart disease. Reports of biochemistry & molecular biology. 2017;6(1):1. 29090223 PMC5643455

[26] Takeda N, Maemura K, Horie S, Oishi K, Imai Y, Harada T, et al. Thrombomodulin is a clock-controlled gene in vascular endothelial cells. Journal of Biological Chemistry. 2007;282(45):32561–7. doi: 10.1074/jbc.M705692200 17848551

[27] Westgate EJ. Cardiovascular rhythms and the molecular clock: University of Pennsylvania; 2007.

[28] Upadhyay KK, Jadeja RN, Vyas HS, Pandya B, Joshi A, Vohra A, et al. Carbon monoxide releasing molecule-A1 improves nonalcoholic steatohepatitis via Nrf2 activation mediated improvement in oxidative stress and mitochondrial function. Redox Biology. 2020;28:101314. doi: 10.1016/j.redox.2019.101314 31514051 PMC6737302

[29] Menghini R, Casagrande V, Cardellini M, Martelli E, Terrinoni A, Amati F, et al. MicroRNA 217 modulates endothelial cell senescence via silent information regulator 1. Circulation. 2009;120(15):1524–32. doi: 10.1161/CIRCULATIONAHA.109.864629 19786632

[30] Shi X, Ma W, Li Y, Wang H, Pan S, Tian Y, et al. MiR-144-5p limits experimental abdominal aortic aneurysm formation by mitigating M1 macrophage-associated inflammation: Suppression of TLR2 and OLR1. Journal of Molecular and Cellular Cardiology. 2020;143:1–14. doi: 10.1016/j.yjmcc.2020.04.008 32278833

[31] Leeper NJ, Raiesdana A, Kojima Y, Chun HJ, Azuma J, Maegdefessel L, et al. MicroRNA‐26a is a novel regulator of vascular smooth muscle cell function. Journal of cellular physiology. 2011;226(4):1035–43. doi: 10.1002/jcp.22422 20857419 PMC3108574

[32] Thounaojam MC, Jadeja RN, Warren M, Powell FL, Raju R, Gutsaeva D, et al. MicroRNA-34a (miR-34a) mediates retinal endothelial cell premature senescence through mitochondrial dysfunction and loss of antioxidant activities. Antioxidants. 2019;8(9):328. doi: 10.3390/antiox8090328 31443378 PMC6769710

[33] Meng F, Glaser SS, Francis H, Yang F, Han Y, Stokes A, et al. Epigenetic regulation of miR-34a expression in alcoholic liver injury. The American journal of pathology. 2012;181(3):804–17. doi: 10.1016/j.ajpath.2012.06.010 22841474 PMC3432440

[34] Gatsiou A, Georgiopoulos G, Vlachogiannis NI, Pfisterer L, Fischer A, Sachse M, et al. Additive contribution of microRNA-34a/b/c to human arterial ageing and atherosclerosis. Atherosclerosis. 2021;327:49–58. doi: 10.1016/j.atherosclerosis.2021.05.005 34038763

[35] Xu Y, Xu Y, Zhu Y, Sun H, Juguilon C, Li F, et al. Macrophage miR-34a is a key regulator of cholesterol efflux and atherosclerosis. Molecular Therapy. 2020;28(1):202–16. doi: 10.1016/j.ymthe.2019.09.008 31604677 PMC6952168

[36] Li T, Pang Q, Liu Y, Bai M, Peng Y, Zhang Z. Sulforaphane protects human umbilical vein endothelial cells from oxidative stress via the miR‑34a/SIRT1 axis by upregulating nuclear factor erythroid‑2‑related factor 2. Experimental and therapeutic medicine. 2021;21(3):1–. doi: 10.3892/etm.2021.9617 33488795 PMC7812584

[37] Guo Y, Xing L, Chen N, Gao C, Ding Z, Jin B. Total flavonoids from the Carya cathayensis Sarg. leaves inhibit HUVEC senescence through the miR‐34a/SIRT1 pathway. Journal of Cellular Biochemistry. 2019;120(10):17240–9. doi: 10.1002/jcb.28986 31106472

[38] Castro RE, Ferreira DM, Afonso MB, Borralho PM, Machado MV, Cortez-Pinto H, et al. miR-34a/SIRT1/p53 is suppressed by ursodeoxycholic acid in the rat liver and activated by disease severity in human non-alcoholic fatty liver disease. Journal of hepatology. 2013;58(1):119–25. doi: 10.1016/j.jhep.2012.08.008 22902550

[39] Zhang F, Zheng W, Wang H. MiR-34a-5p inhibition attenuates LPS-induced endothelial cell injury by targeting FOXM1. Eur Rev Med Pharmacol Sci. 2020;24(20):10829–38. doi: 10.26355/eurrev_202010_23445 33155244

[40] Cui G-m, Zhao Y-x, Zhang N-n, Liu Z-s, Sun W-c, Peng Q-s. Amiloride attenuates lipopolysaccharide-accelerated atherosclerosis via inhibition of NHE1-dependent endothelial cell apoptosis. Acta Pharmacologica Sinica. 2013;34(2):231–8. doi: 10.1038/aps.2012.155 23274414 PMC4011619

[41] Stein S, Matter CM. Protective roles of SIRT1 in atherosclerosis. Cell cycle. 2011;10(4):640–7. doi: 10.4161/cc.10.4.14863 21293192

[42] Zhang H, Zhao Z, Pang X, Yang J, Yu H, Zhang Y, et al. MiR-34a/sirtuin-1/foxo3a is involved in genistein protecting against ox-LDL-induced oxidative damage in HUVECs. Toxicology Letters. 2017;277:115–22. doi: 10.1016/j.toxlet.2017.07.216 28688900

[43] Jun S, Datta S, Wang L, Pegany R, Cano M, Handa JT. The impact of lipids, lipid oxidation, and inflammation on AMD, and the potential role of miRNAs on lipid metabolism in the RPE. Experimental eye research. 2019;181:346–55. doi: 10.1016/j.exer.2018.09.023 30292489 PMC6443454

[44] Mehana K. Cell Cycle Regulation of Stem Cells by MicroRNAs. 2020. 10.1007/s12015-018-9808-y PMC5960494 29541978

[45] Vyas HS, Upadhyay KK, Devkar RV. miRNAs Signatures In Patients With Acute Liver Injury: Clinical Concerns and Correlations. Current Molecular Medicine. 2020;20(5):325–35. doi: 10.2174/1566524020666191211153546 31823701

[46] Ma Y, Chai N, Jiang Q, Chang Z, Chai Y, Li X, et al. DNA methyltransferase mediates the hypermethylation of the microRNA 34a promoter and enhances the resistance of patient-derived pancreatic cancer cells to molecular targeting agents. Pharmacological Research. 2020;160:105071. doi: 10.1016/j.phrs.2020.105071 32659427

[47] Rezaei M, Eskandari F, Mohammadpour-Gharehbagh A, Harati-Sadegh M, Teimoori B, Salimi S. Hypomethylation of the miRNA-34a gene promoter is associated with Severe Preeclampsia. Clinical and Experimental Hypertension. 2019;41(2):118–22. 10.1080/10641963.2018.1451534 29557690

[48] Doridot L, Houry D, Gaillard H, Chelbi ST, Barbaux S, Vaiman D. miR-34a expression, epigenetic regulation, and function in human placental diseases. Epigenetics. 2014;9(1):142–51. doi: 10.4161/epi.26196 24081307 PMC3928177

[49] Yang M, Lee J-E, Padgett RW, Edery I. Circadian regulation of a limited set of conserved microRNAs in Drosophila. BMC genomics. 2008;9(1):1–11. doi: 10.1186/1471-2164-9-83 18284684 PMC2263044

[50] Zhu J-N, Fu Y-H, Hu Z-q, Li W-Y, Tang C-M, Fei H-W, et al. Activation of miR-34a-5p/Sirt1/p66shc pathway contributes to doxorubicin-induced cardiotoxicity. Scientific reports. 2017;7(1):1–12. 28928469 10.1038/s41598-017-12192-y PMC5605522

