# Supplementary figures and images for: miR34a-5p impedes CLOCK expression in chronodisruptive C57BL/6J mice and potentiates pro-atherogenic manifestations
Source: PLoS One. 2023 Aug 10;18(8):e0283591. doi: 10.1371/journal.pone.0283591 (PMC10414636; doi:10.1371/journal.pone.0283591)

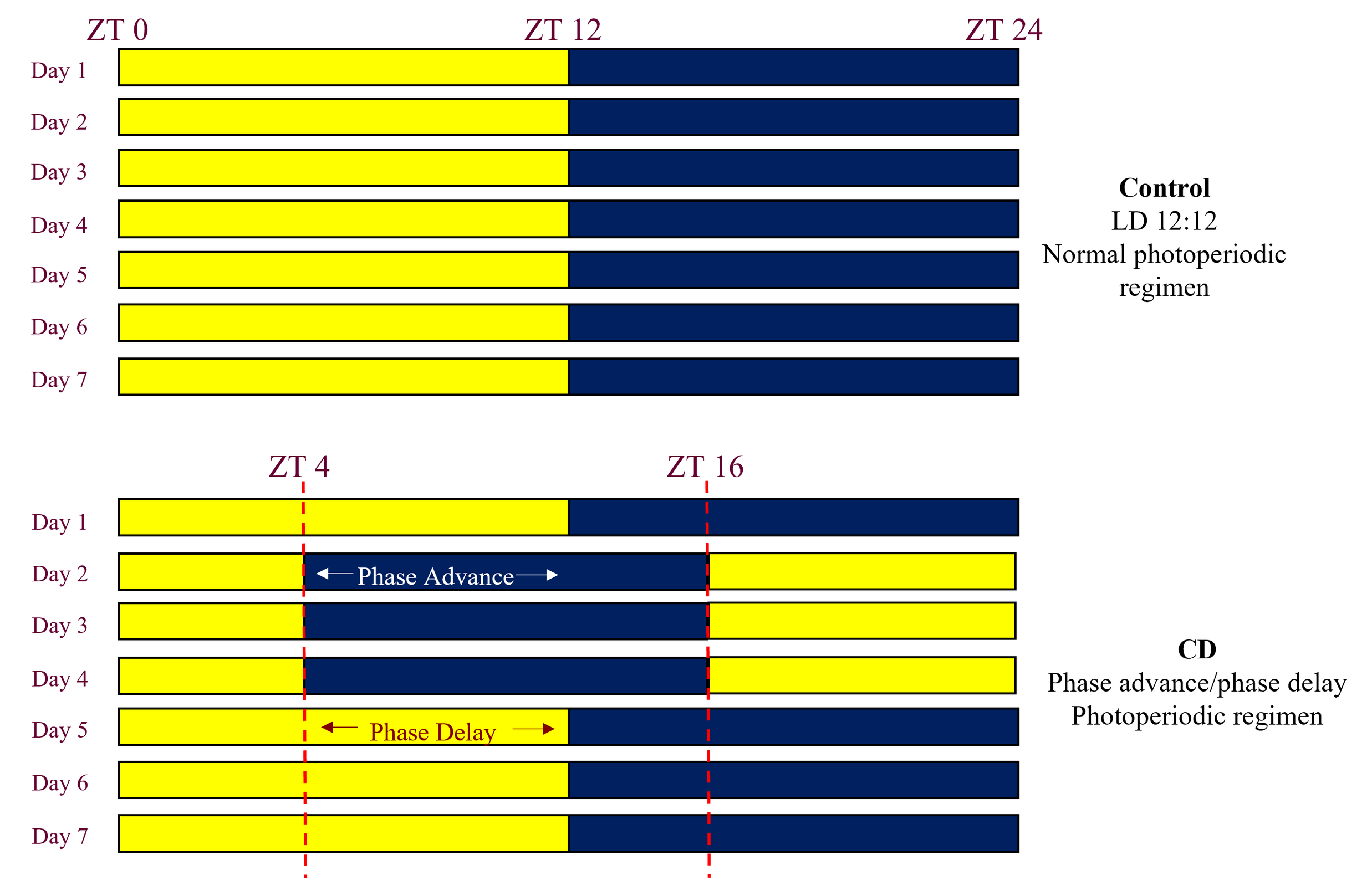

Supplement: S1 Fig — Control C57BL/6J mice were subjected to LD 12:12. Whereas CD mice were subject to 8h phase advance from day 2 and 8h phase delay from day 5 for 18 weeks. (TIF) [file pone.0283591.s001.tif]

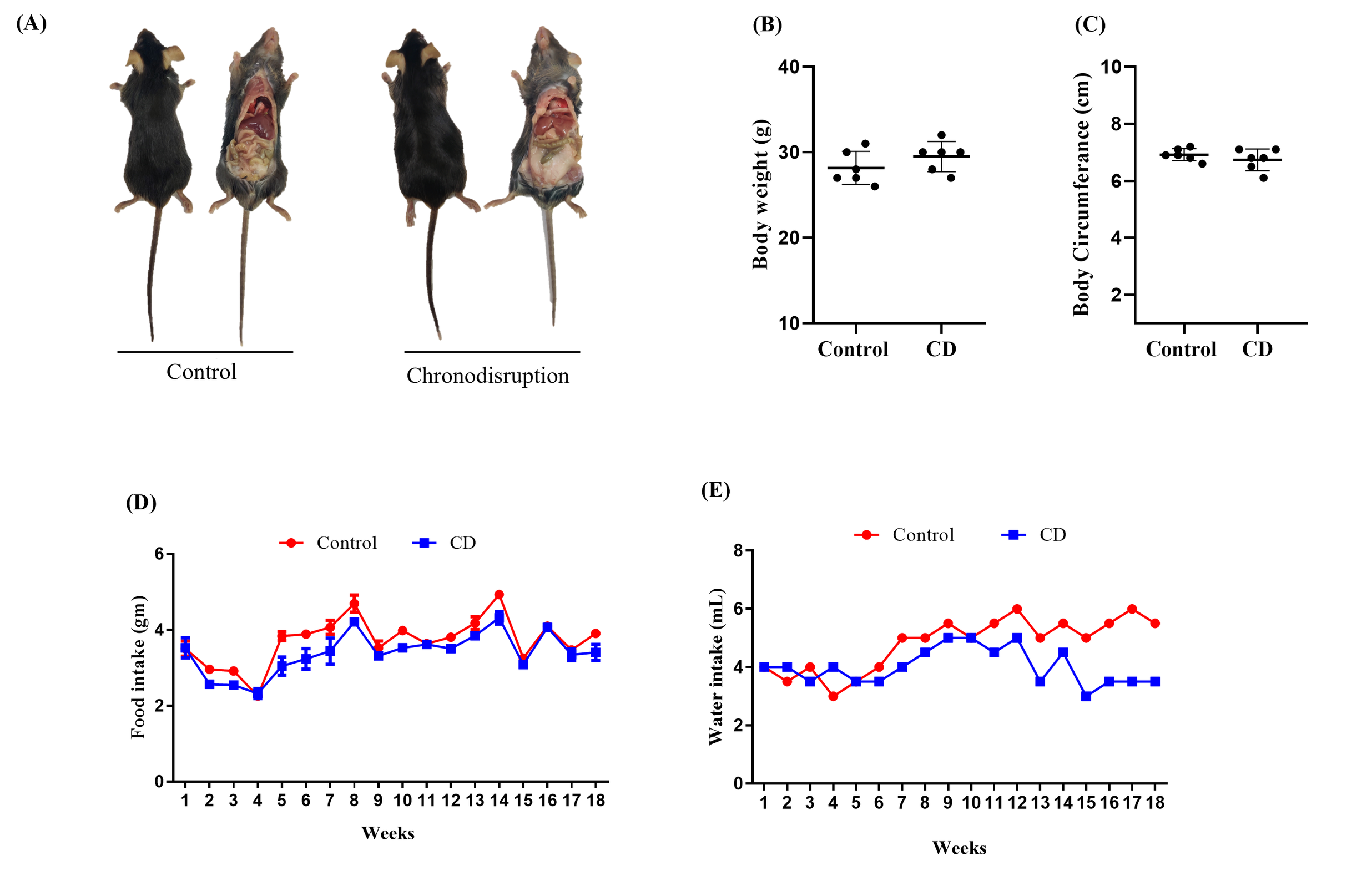

Supplement: S2 Fig — (A) Biopsy of C57BL/6J mice. (B) Body weight (C) Body Circumference recorded at the end of experiment; (D) Food intake and (E) water intake recorded throughout the period of study in Control and CD mice. (TIF) [file pone.0283591.s002.tif]

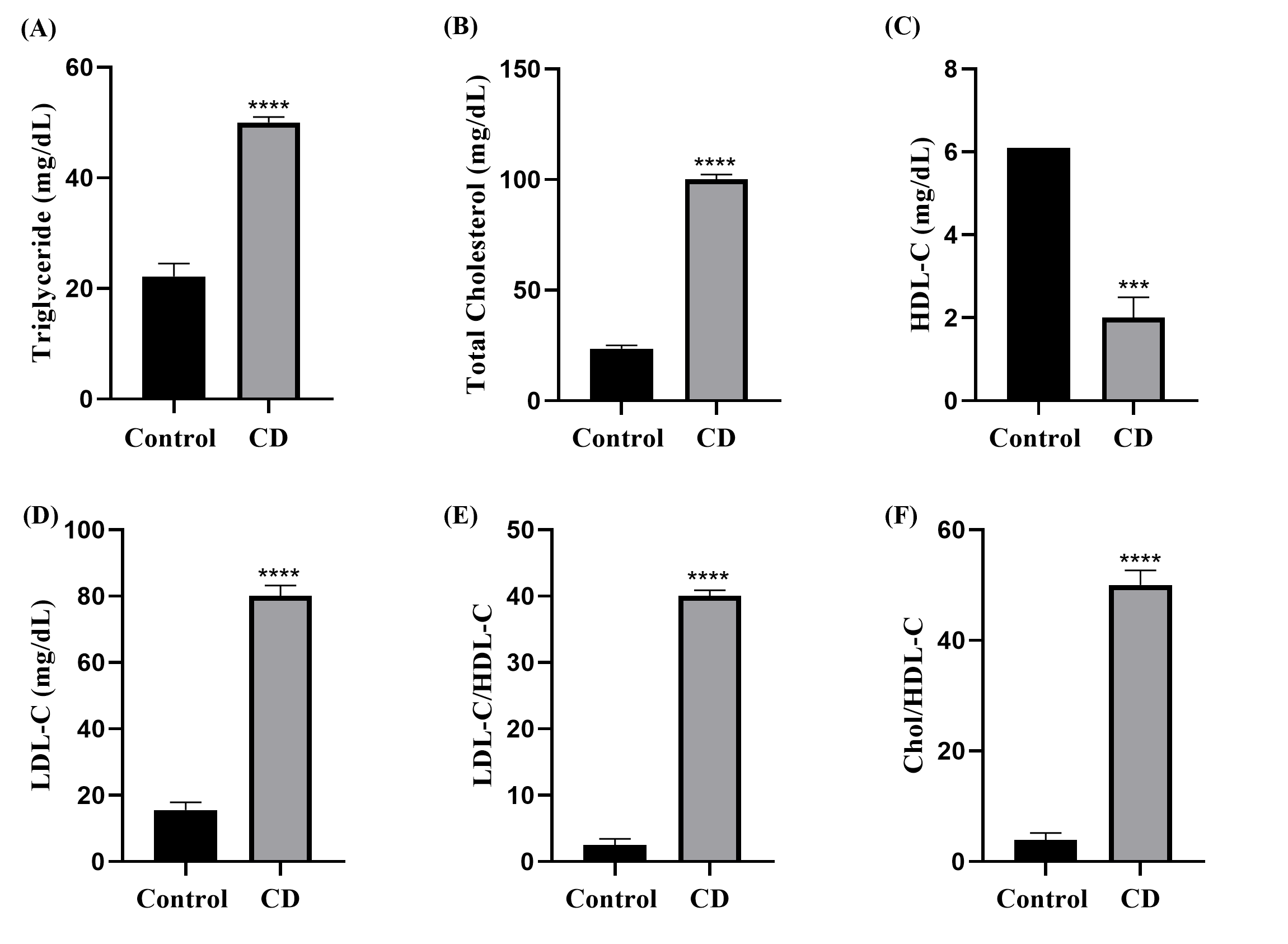

Supplement: S3 Fig — Results are expressed as mean ± SD *p < 0.05, **p < 0.01 or ***p < 0.001 for CD vs control group. (TIF) [file pone.0283591.s003.tif]

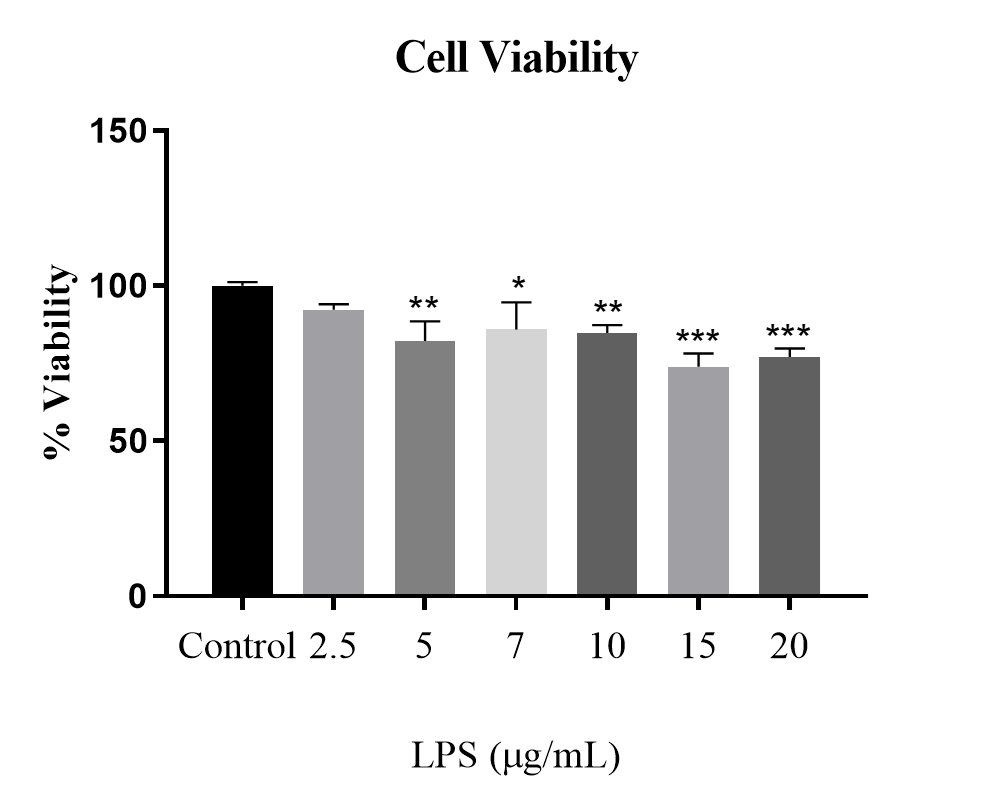

Supplement: S4 Fig — Data represents % viable cells at different concentrations. (TIF) [file pone.0283591.s004.tif]
