# Supplementary material for: miR34a-5p impedes CLOCK expression in chronodisruptive C57BL/6J mice and potentiates pro-atherogenic manifestations
Source: PLoS One. 2023 Aug 10;18(8):e0283591. doi: 10.1371/journal.pone.0283591 (PMC10414636; doi:10.1371/journal.pone.0283591)
Supplement: S1 Table — Also shown are the dock scores and seed regions of miR34a-5p and, promoter length of clock gene in vertebrates viz. mice, rat and human. (DOCX) [file pone.0283591.s005.docx]

**S1 Table**

| **Species** | **Gene** | **Total no. of miRNAs docking to clock** | **miRNA** | **Dock Score** | **Seed location** | **Length of promoter region** |
| --- | --- | --- | --- | --- | --- | --- |
| *Homo sapiens* | *Clock* | 628 | hsa-miR 34a-5p | 90 | 389,  5954 | 7514 nt |
| *Mus musculus* | *Clock* | 397 | mmu-miR 34a-5p | 58 | 369 | 6845 nt |
| *Rattus norvegicus* | *Clock* | 163 | rno-miR 34a-5p | 64 | 363 | 6702 nt |
