# Supplementary material for: miR34a-5p impedes CLOCK expression in chronodisruptive C57BL/6J mice and potentiates pro-atherogenic manifestations
Source: PLoS One. 2023 Aug 10;18(8):e0283591. doi: 10.1371/journal.pone.0283591 (PMC10414636; doi:10.1371/journal.pone.0283591)
Supplement: S2 Table — (DOCX) [file pone.0283591.s006.docx]

**S2 Table**

| **Sl. No.** | **Name** | **Fwd Primer (5’-3’)** | **Rev Primer (5’-3’)** |
| --- | --- | --- | --- |
| 1 | hsa-miR-34a-5p | CGAGTGGCAGTGTCTTAGCT | CCAGTTTTTTTTTTTTTTTTTACAACC |
| 2 | hsa-*5S* | GGCCATACCACCCTGAACGC | CAGCACCCGGTATTCCCAGG |
| 3 | hsa-*18s rRNA* | CGTTCAGCCACCCGAGATT | GACCCGCACTTACTGGGAATT |
| 4 | hsa-*Clock* | CGAGCGCTCCCGAATTTTTA | AGGTATCTAGTGAGACTTGCCA |
| 5 | hsa-*Bmal1* | GGCTCATAGATGCAAAAACTGG | CTCCAGAACATAATCGAGATGG |
| 6 | hsa-*Per2* | GACTCCTCGGCTTGAAACGG | GTGTCACCGCAGTTCAAACG |
| 7 | hsa-*Cry2* | GTGCCTCAAATCCTGACCCA | GCCTCCCACAAGATTGACGA |
| 8 | mmu-miR34-5p | GCAGTGGCAGTGTCTTAG | GGTCCAGTTTTTTTTTTTTTTTACAAC |
| 9 | mmu-*5S* | TCTCGTCTGATCTCGGAAGC | AGCCTACAGCACCCGGTATT |
| 10 | mmu-*18s rRNA* | GCAATTATTCCCCATGAACG | GGCCTCACTAAACCATCCAA |
| 11 | mmu- *Clock* | CACTCTCACAGCCCCACTGTA | CCCCACAAGCTACAGGAGCAG |
| 12 | mmu- *Bmal1* | ACATAGGACACCTCGCAGAA | AACCATCGACTTCGTAGCGT |
| 13 | mmu-*Per1* | CATGACTGCACTTCGGGAGC | CTTGACACAGGCCAGAGCGTA |
| 14 | mmu-*Per2* | GGCTTCACCATGCCTGTTGT | GGAGTTATTTCGGAGGCAAGTGT |
| 15 | mmu-*Cry2* | TCGGCTCAACATTGAACGAA | TCGGCTCAACATTGAACGAA |
| 16 | mmu-*Sirt1* | GATACCTTGGAGCAGGTTGC | CTCCACGAACAGCTTCACAA |
