# Supplementary material for: miR34a-5p impedes CLOCK expression in chronodisruptive C57BL/6J mice and potentiates pro-atherogenic manifestations
Source: PLoS One. 2023 Aug 10;18(8):e0283591. doi: 10.1371/journal.pone.0283591 (PMC10414636; doi:10.1371/journal.pone.0283591)

Western Blots:

Figure1 (B)

Control: C

Chronodisruption: CD

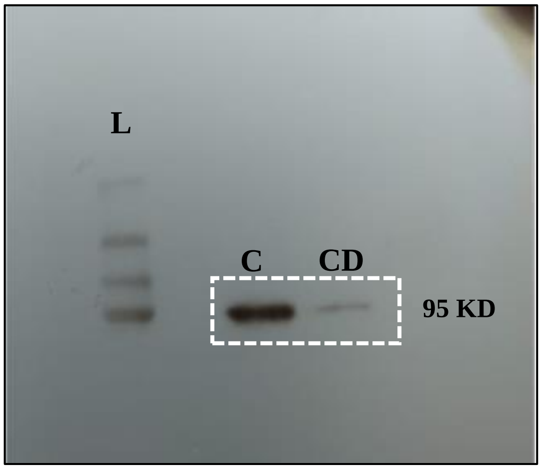

CLOCK (Thoracic Aorta)

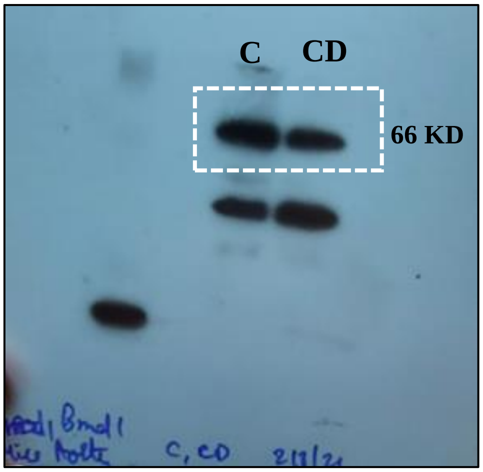

BMAL1 (Thoracic Aorta)

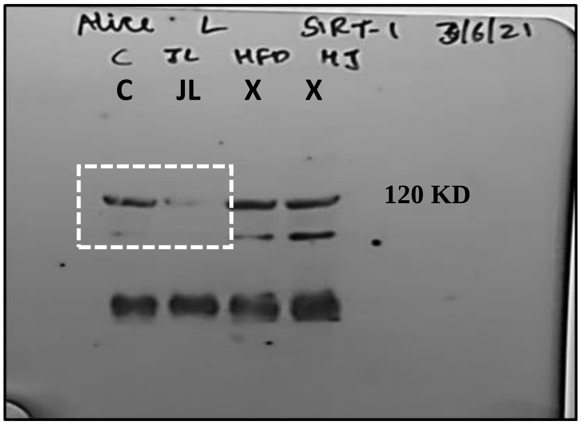

SIRT-1 (Thoracic Aorta)

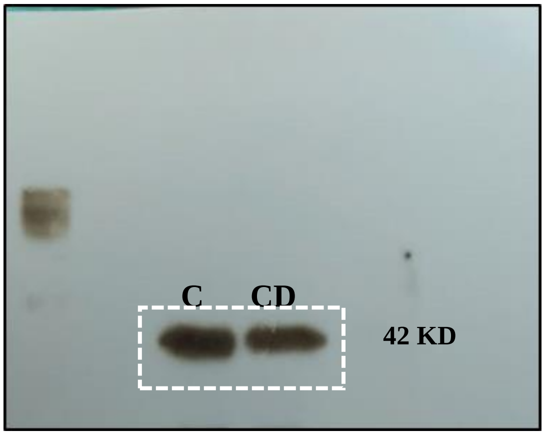

β-Actin (Thoracic Aorta)

Figure 4 (C)

Control: C

LPS Treatment: LPS

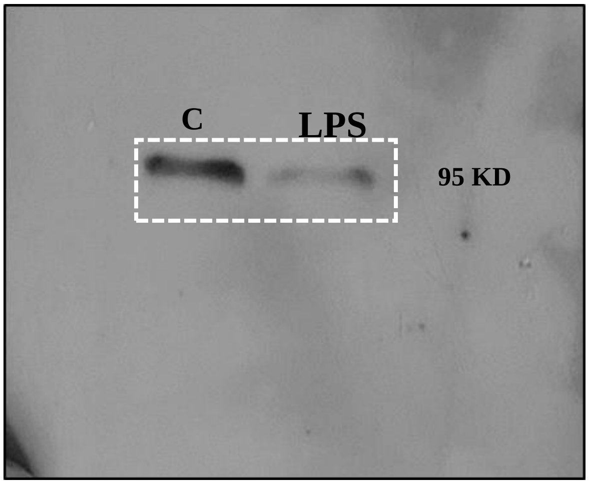

CLOCK (HUVEC)

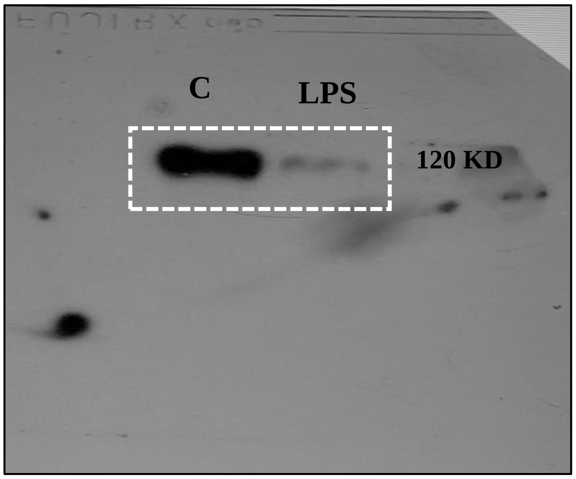

SIRT-1 (HUVEC)

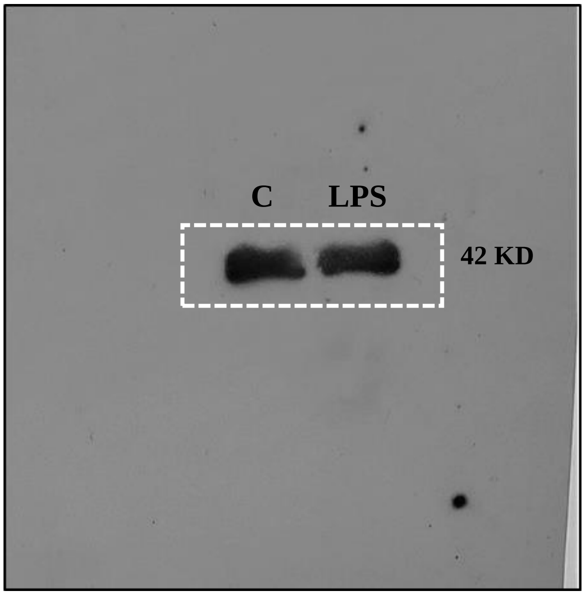

β-Actin (HUVEC)

Figure 5 (B)

Time point based (ZT 0, 6, 12, 18, 24) protein assessment from thoracic aorta of C57BL/6J mice.

ZT 0 6 12 18 24

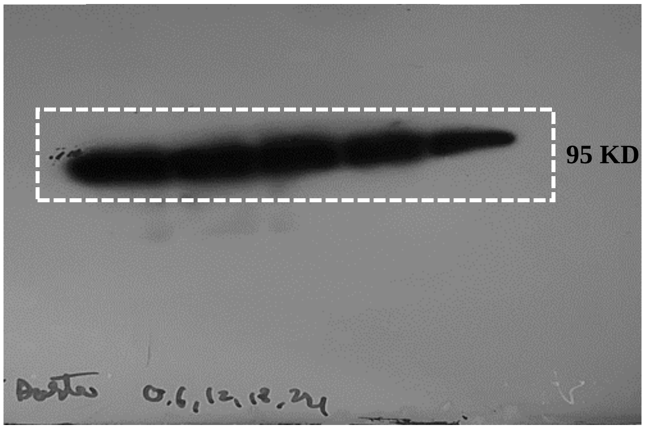

CLOCK (Thoracic Aorta)

ZT 0 6 12 18 24

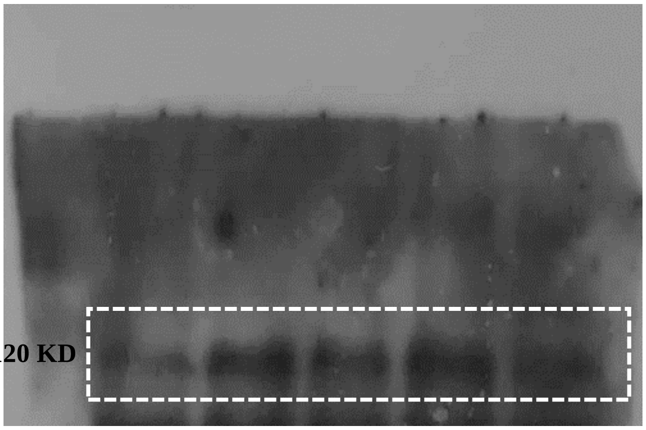

SIRT-1 (Thoracic Aorta)

ZT 0 6 12 18 24

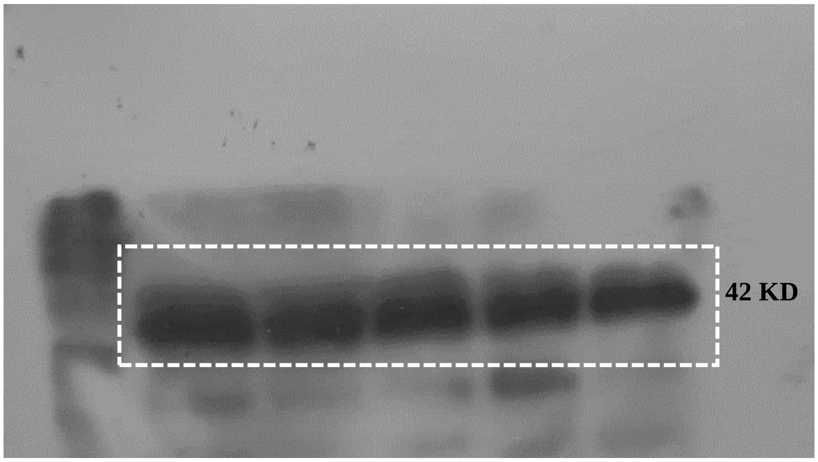

β-Actin (Thoracic Aorta)

Figure 5 (D)

Time point based (ZT 0, 6, 12, 18, 24) protein assessment from synchronized HUVEC cells.

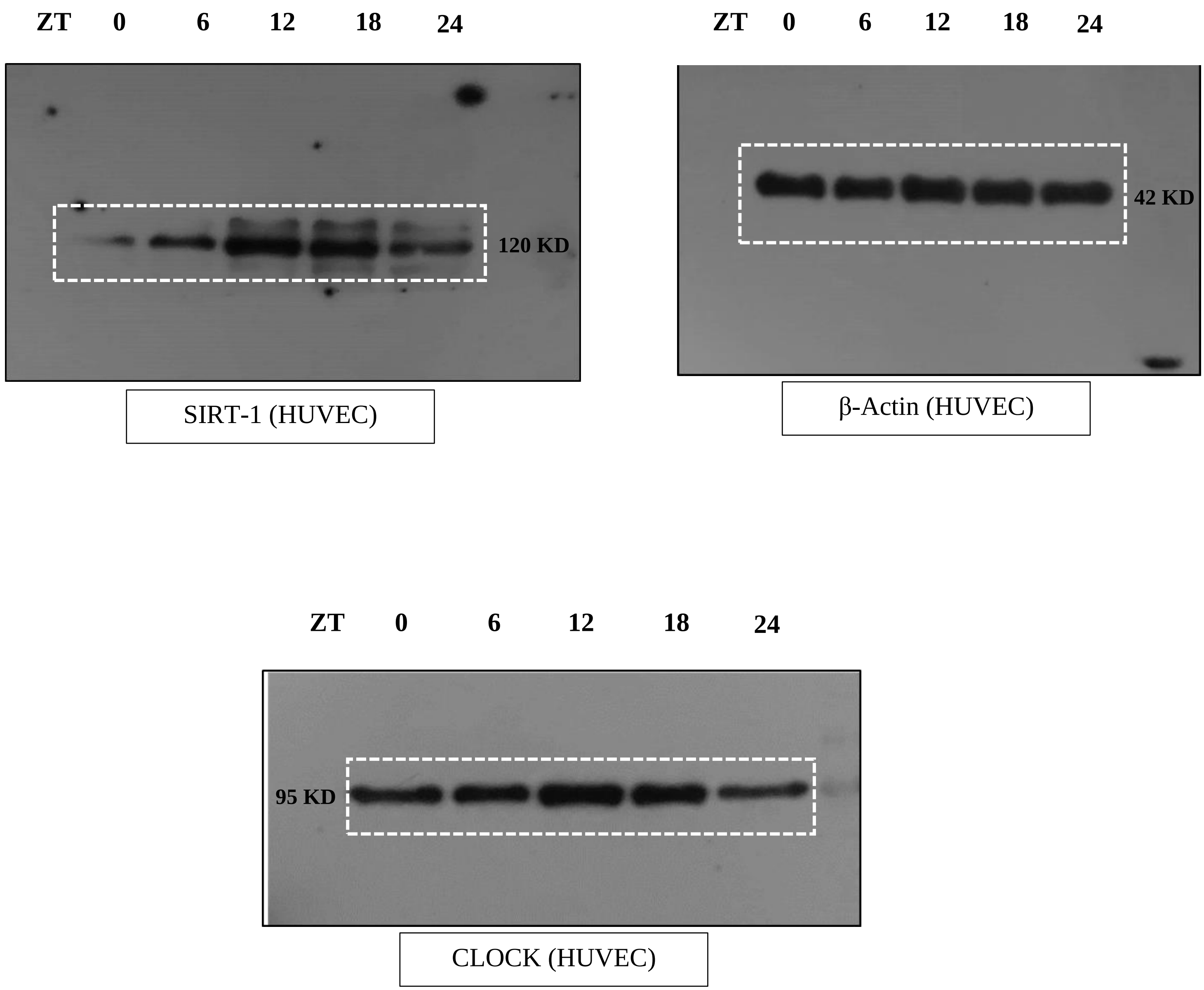

Supplement: S1 Raw images — (PDF) [file pone.0283591.s007.pdf]
